# Compound heterozygous mutations in the noncoding *RNU4ATAC* cause Roifman Syndrome by disrupting minor intron splicing

**DOI:** 10.1038/ncomms9718

**Published:** 2015-11-02

**Authors:** Daniele Merico, Maian Roifman, Ulrich Braunschweig, Ryan K. C. Yuen, Roumiana Alexandrova, Andrea Bates, Brenda Reid, Thomas Nalpathamkalam, Zhuozhi Wang, Bhooma Thiruvahindrapuram, Paul Gray, Alyson Kakakios, Jane Peake, Stephanie Hogarth, David Manson, Raymond Buncic, Sergio L. Pereira, Jo-Anne Herbrick, Benjamin J. Blencowe, Chaim M. Roifman, Stephen W. Scherer

**Affiliations:** 1 The Centre for Applied Genomics (TCAG), Program in Genetics and Genome Biology, The Hospital for Sick Children, Toronto, Ontario, Canada M5G 0A4; 2 Division of Clinical and Metabolic Genetics, Department of Paediatrics, The Hospital for Sick Children, Toronto, Ontario, Canada M5G 1X8; 3 The Prenatal Diagnosis and Medical Genetics Program, Department of Obstetrics and Gynaecology, Mount Sinai Hospital, Toronto, Ontario, Canada M5G 1Z5; 4 Department of Paediatrics, University of Toronto, Toronto, Ontario, Canada M5G 1X8; 5 Donnelly Centre, University of Toronto, Toronto, Ontario, Canada M5S 3E1; 6 Division for Immunology and Allergy, Canadian Center for Primary Immunodeficiency, The Hospital for Sick Children, Toronto, Ontario, Canada M5G 1X8; 7 Department of Immunology and Infectious Diseases, Sydney Children's Hospital, Sydney, New South Wales 2031, Australia; 8 Department of Allergy and Immunology, The Children's Hospital at Westmead, Westmead, New South Wales 2145, Australia; 9 Queensland Paediatric Immunology and Allergy Service, The Lady Cilento Children's Hospital, South Brisbane, Queensland 4101, Australia; 10 School of Medicine, University of Queensland, Brisbane, Queensland 4006, Australia; 11 Department of Diagnostic Imaging, The Hospital for Sick Children, Toronto, Ontario, Canada M5G 1X8; 12 Department of Ophthalmology and Vision Sciences, The Hospital for Sick Children, Toronto, Ontario, Canada M5G 1X8; 13 Department of Molecular Genetics, University of Toronto, Toronto, Ontario, Canada M5S 1A8; 14 McLaughlin Centre, University of Toronto, Toronto, Ontario, Canada M5G 0A4; 15 Centre of Excellence in Genomic Medicine Research (CEGMR), King Abdulaziz University, Jeddah 21589, Kingdom of Saudi Arabia

## Abstract

Roifman Syndrome is a rare congenital disorder characterized by growth retardation, cognitive delay, spondyloepiphyseal dysplasia and antibody deficiency. Here we utilize whole-genome sequencing of Roifman Syndrome patients to reveal compound heterozygous rare variants that disrupt highly conserved positions of the *RNU4ATAC* small nuclear RNA gene, a minor spliceosome component that is essential for minor intron splicing. Targeted sequencing confirms allele segregation in six cases from four unrelated families. *RNU4ATAC* rare variants have been recently reported to cause microcephalic osteodysplastic primordial dwarfism, type I (MOPD1), whose phenotype is distinct from Roifman Syndrome. Strikingly, all six of the Roifman Syndrome cases have one variant that overlaps MOPD1-implicated structural elements, while the other variant overlaps a highly conserved structural element not previously implicated in disease. RNA-seq analysis confirms extensive and specific defects of minor intron splicing. Available allele frequency data suggest that recessive genetic disorders caused by *RNU4ATAC* rare variants may be more prevalent than previously reported.

## Abstract (web summary)

Roifman Syndrome is a rare disorder whose disease manifestations include growth retardation, spondyloepiphyseal dysplasia and immunodeficiency. Here, the authors use whole-genome sequencing to discover that rare compound heterozygous variants disrupting the small nuclear RNA gene *RNU4ATAC* cause Roifman Syndrome.

## Graphical abstract

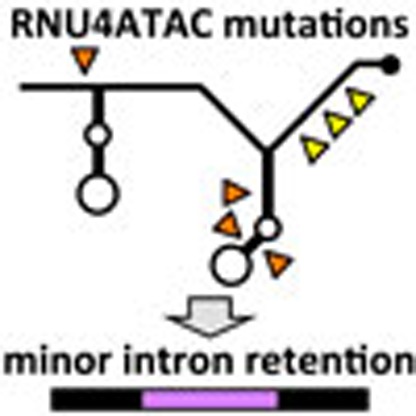

Roifman Syndrome (OMIM 300258) was first described as a novel association of antibody deficiency, spondyloepiphyseal chondro-osseous dysplasia, retinal dystrophy, poor pre- and postnatal growth, cognitive delay and facial dysmorphism^1^,^2^. In spite of some variability, all subjects share remarkably identical dysmorphic, skeletal and immunological features^1^,^2^,^3^,^4^,^5^,^6^.

It was proposed that Roifman Syndrome might be a novel ciliopathy with immunodeficiency, because of retinal dystrophy and some early and transient bone changes^7^. X-linked inheritance was also suspected because most reported cases were males^1^,^2^,^7^. Candidate gene studies using targeted sequencing were unsuccessful in identifying causal variants.

Here we applied whole-genome sequencing in two affected siblings and exhaustive analysis of coding as well as noncoding variants. We identified rare compound heterozygous variants disrupting highly conserved elements of the small nuclear RNA (snRNA) gene *RNU4ATAC* (RefSeq NR_023343, OMIM 601428), which is essential for minor intron splicing^8^,^9^,^10^,^11^ and was reported to cause the recessive disorder microcephalic osteodysplastic primordial dwarfism, type I (MOPD1, OMIM 210710) (refs 10, 11). Roifman Syndrome is phenotypically distinct from MOPD1 and presents a unique pattern of compound heterozygosity, which was confirmed in four unrelated families by targeted sequencing. About 800 genes have one (or less often more than one) minor intron and thus are dependent on the minor spliceosome for correct splicing^9^. Since they are involved in important cellular functions (DNA repair and replication, transcription, RNA processing, cell cycle, etc.) their incorrect splicing can alter cell functionality and viability. RNA-seq analysis confirmed specific alterations of minor intron splicing in Roifman Syndrome patients. In addition, we integrated RNA-seq results with other phenotypic evidence to prioritize genes whose splicing alteration is more likely implicated in Roifman Syndrome.

## Results

### Clinical features of Roifman Syndrome patients

**Figure 1:**
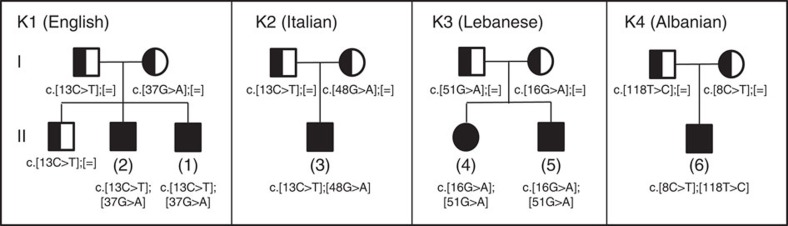
Pedigree of the six Roifman Syndrome cases (kindred 1–4). The pedigrees show the *RNU4ATAC* compound heterozygous SNVs in the six genotyped cases of Roifman Syndrome; [=] indicates no variant detected.

We assembled almost all available Roifman Syndrome-affected subjects (six from four unrelated families) for this study (Fig. 1; see Supplementary Tables 1 and 2 and Supplementary Note 1 for a detailed subject description).

**Figure 2:**
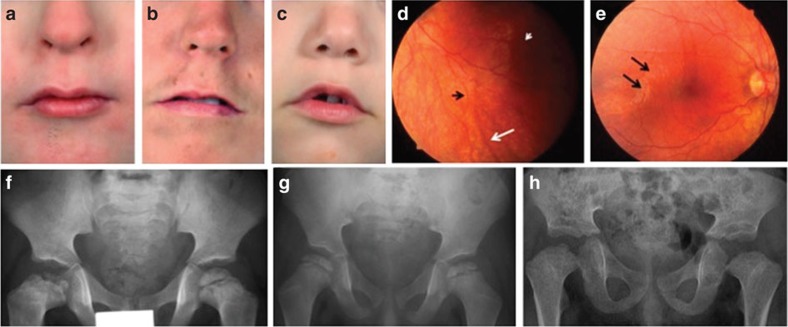
Facial, retinal and skeletal features of Roifman Syndrome. Facial dysmorphic features (**a**–**c**) include a markedly long philtrum, thin upper lip and down-turned corners of the mouth. Retinal features are displayed for patient 3 at age 4 years: arteriolar attenuation (**d**, black arrow), wrinkling of the inner limiting membrane (**d**, short white arrow and **e**, black arrows) and pigmentary changes (**d**, long white arrow). Skeletal features are displayed for patients 1, 3 and 5, respectively: the radiographs show the proximal epiphyses of the femora with symmetric delayed ossification, as well as flattening and irregularity.

All individuals shared the following facial features: a markedly long philtrum with a thin upper lip (Fig. 2), a narrow, tubular and upturned nose with hypoplastic alae nasi (Fig. 2), widely spaced eyes with long palpebral fissures and prominent lashes.

The six cases also presented highly characteristic skeleton and immune abnormalities. The proximal epiphyses of the femora demonstrated symmetric delayed ossification, as well as mild flattening and irregularity (Fig. 2); unlike Schimke immune-osseous dysplasia, the acetabulae were normal. Similar but less pronounced changes could be seen in the other epiphyses of the axial skeleton: the vertebrae were ‘bullet' shaped or biconvex at an age one would expect them to be ‘squarer'. In addition, all six cases had brachydactyly, while four had transverse palmar creases and clinodactyly of the fifth digit.

While serum immunoglobin levels were variable, all patients were unable to produce specific antibodies. Circulating B-cell number was on the lower end of the normal range, with mature B cell and memory B-cell numbers within normal ranges. T-cell number and function were completely normal (see Supplementary Table 2 for detailed immunological findings).

Finally, three of six patients had retinal dystrophy with extensive degeneration of the rod and cone systems.

### Whole-genome and targeted sequencing results

We applied whole-genome sequencing of two affected individuals from kindred 1, to search for putative causal variants in an unbiased and hypothesis-free manner. Variants were prioritized based on sequencing quality, allele frequency in reference databases below 1%, gene product damage potential, zygosity and gene mode of inheritance.

None of the two siblings had any high-quality, rare, damaging homozygous variants.

No X-linked variant passing the prioritization criteria was shared. However, since the X-linked mode of inheritance had been proposed for Roifman Syndrome, we additionally investigated X-chromosome variants found in only one of the two siblings; they either did not have any known implication in human genetic disorders or mouse abnormal phenotypes (genes *ARSH* and *HS6ST2-AS1*), or they had modest protein-damaging potential and insufficient match to Roifman Syndrome phenotype (genes *AFF2* and *SH3KBP1*, for more details see Supplementary Data 1, Supplementary Tables 3–5 and Supplementary Note 2).

While a dominant mode of inheritance was highly unlikely, we investigated three high-quality variants with very rare allele frequency (<0.1%) impacting genes with a dominant mode of inheritance and shared by the two siblings (*GUCY2D*, *HTT* and *RP1L1*); unsurprisingly, on more detailed review, we found insufficient match to the Roifman Syndrome phenotype and only modest damaging potential (for more details see Supplementary Data 1, Supplementary Table 5 and Supplementary Note 2). Copy number and structural variant findings were also negative (see Supplementary Data 2 and 3 and Supplementary Note 2).

Finally, we reviewed genes with more than one heterozygous variant, thus potentially consisting of a compound heterozygous set. Only one set passed the prioritization criteria and was shared between the two siblings (Supplementary Table 5), corresponding to two heterozygous single-nucleotide substitutions in the autosomal *RNU4ATAC* gene encoding the highly conserved U4atac snRNA, an essential component of the minor spliceosome^8^,^9^,^10^,^11^. Compound heterozygous variants clustering in similar *RNU4ATAC* structural elements were subsequently identified by Sanger sequencing in four other Roifman syndrome-affected patients from three ethnically different families (Fig. 1 and Table 1). Analysis of more than 800 whole genome sequences with unrelated conditions revealed no homozygosity or potential compound heterozygosity at disease variant loci or other conserved positions (Supplementary Note 3).

### *RNU4ATAC* in MOPD1 and Roifman Syndrome

*RNU4ATAC* homozygous and compound heterozygous variants were recently reported to cause the recessive disorder MOPD1 (OMIM 210710) (refs 10, 11). The lethality of MOPD1 revealed the importance of the minor spliceosome, which is required for correct splicing of about 800 human genes carrying minor introns, including many genes involved in essential functions^9^,^10^,^11^. MOPD1 and recessive isolated familial growth hormone deficiency (caused by compound heterozygous variants in the *RNPC3* protein-coding gene)^12^ are the only known congenital disorders caused by the disruption of a minor spliceosome-specific component, that is, not present in the major spliceosome (for more details see Supplementary Note 4).

Typical MOPD1 is clearly distinguishable from Roifman Syndrome, as it is characterized by pre- and postnatal lethality, severe prenatal microcephaly and brain malformations, intractable epilepsy, short and bowed limbs, absent or sparse hair, neuroendocrine dysfunction and distinct facial features including proptotic eyes, large/prominent nose or downturned nasal tip and micrognathia^13^,^14^ (Table 2). Skin and retinal hypopigmentation have been described in a few cases^15^. Patients with a milder phenotype of MOPD1 have also been recently described^15^,^16^,^17^,^18^; they exhibit severe microcephaly, with poorly developed gyri and agenesis of corpus callosum, and typical dysmorphic features (striking micrognathia, absent eye brows, large prominent nose, dysplastic nails and, in some, agenesis of ear lobes). These features are not shared with Roifman Syndrome. Skeletal changes and eye pigmentation abnormalities in MOPD1 cases are also different from the epiphyseal dysplasia and severe retinal dysplasia typical of Roifman Syndrome. Finally, none of the MOPD1 cases, severe or mild, were reported to have evidence of primary immunodeficiency (Table 2).

### Variant impact on *RNU4ATAC* structural elements

The *RNU4ATAC* structural elements perturbed in MOPD1 and Roifman Syndrome suggest a molecular basis for the phenotypic differences between the two syndromes.

**Figure 3:**
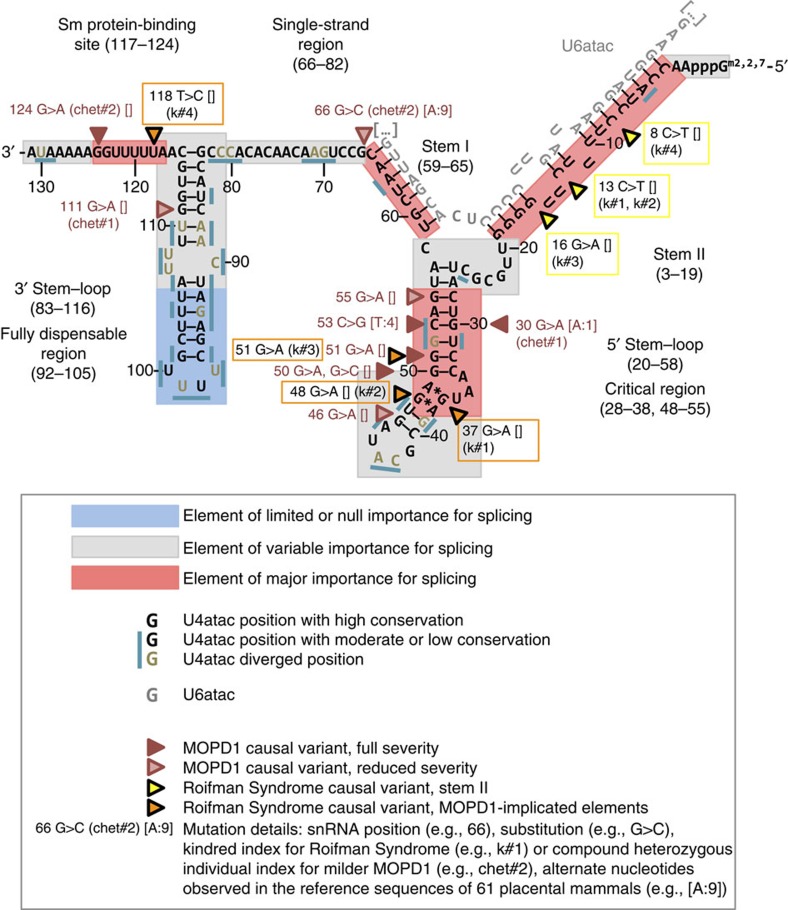
U4atac snRNA secondary structure elements, positional conservation, MOPD1 and Roifman Syndrome causal variants. Elements of limited or null importance for splicing (dispensable region of 3′ stem–loop) have mutagenesis experiments^20^,^21^ showing no splicing alteration, are enriched in low conservation and do not present any known disease-causing variant. Elements of variable importance for splicing (3′ stem–loop, except dispensable region; single-strand region; 5′ stem–loop, except critical region) have mutagenesis experiments showing modest or no splicing alteration, or have not been probed by mutagenesis, but meet at least one of these criteria: (a) they present at least one MOPD1 causal variant (typically with reduced severity); (b) they are proximal to a MOPD1 causal variant cluster; (c) structural studies^60^,^61^ suggest they may have a functional role; these elements have mixed conservation, and only a few variants at more conserved positions may cause splicing alterations. Elements of major importance for splicing (stem II, critical region of the 5′ stem–loop, stem I, Sm protein-binding site) have mutagenesis experiments producing splicing alterations and/or overlap the MOPD1 variant cluster; in addition, they are expected to have a major functional role based on structural studies; finally, they are enriched in highly conserved positions, the majority of which are expected to cause splicing alterations in presence of variation. Positions are labelled as ‘high conservation' if placental mammal or 100-vertebrate PhyloP ≥1.75, as ‘diverged' if placental mammal and 100-vertebrate PhyloP are negative, and ‘moderate or low conservation' otherwise. Parts of U6atac are displayed only in correspondence of U4atac–U6atac duplex structures. U4atac snRNA coordinate 1 corresponds to hg19 coordinate 122,288,456. Classification of MOPD1 causal variants as ‘full severity' or ‘reduced severity' is based on a thorough review of MOPD1 literature^10^,^11^,^15^,^16^,^17^,^18^ (for more details, see Supplementary Table 17) and biochemical assays of variant effect^22^; the latter is particularly important for less severe MOPD1 forms presenting compound heterozygosity.

The U4atac snRNP (snRNA ribonucleoprotein) is required for the formation of the catalytically active minor spliceosomal complex, by loading U6atac onto the U12-containing pre-spliceosomal complex in concert with the U5 snRNP^19^ (Supplementary Fig. 1). Regions of U4atac that base pair with U6atac, the stem I and stem II (at the 3′ and 5′ of U4atac, respectively), are separated by an intramolecular stem–loop (the 5′ stem–loop). Another stem–loop is present at the 3′ end of U4atac, followed by a sequence acting as binding site for Sm proteins, which are important for snRNP assembly and import into the nucleus. The stem II, the 5′ stem–loop, the stem I and the Sm-binding site are all enriched in highly conserved nucleotides and mutagenesis experiments have demonstrated their importance for correct minor spliceosomal activity, while part of the 3′ stem is fully dispensable, and other sequence elements have lower conservation^20^,^21^ (Fig. 3, see also Supplementary Figs 2 and 3).

Most MOPD1 causal variants cluster in the 5′ stem–loop (U4atac snRNA positions 30, 46 and 50–55), while only a few are found at other elements (U4atac snRNA position 66, connecting the single-strand region to stem I; position 111, part of the 3′ stem–loop; position 124, part of the Sm-binding site)^10^,^11^,^15^,^16^,^17^,^18^ (Fig. 3). In contrast, all Roifman Syndrome causal variants identified in the six cases are always compound heterozygous (Fig. 1 and Table 1), with one variant overlapping the MOPD1-implicated 5′ stem–loop critical region (U4atac snRNA positions 37, 48 and 51) or the Sm protein-binding site (U4atac snRNA position 118), while the other variant occurs at highly conserved positions in the stem II (U4atac snRNA positions 8, 13 and 16; Fig. 3). On the basis of the secondary structure and conservation profile of U4atac, we expect the Roifman Syndrome causal variants occurring in the 5′ stem–loop to have a destabilizing effect similar to MOPD1 variants. The alteration of the stem II is, however, unique to Roifman Syndrome (Fig. 3). While MOPD1 5′ stem–loop variants impair binding of the *NHP2L1* and *PRPF31* proteins, stem II variants may affect the PRPF4/PRPF3/PPIH protein complex binding in this region^22^. Considering the greater severity of MOPD1, it is reasonable to hypothesize that these stem II variants have a weaker effect on minor spliceosome function.

### RNA-seq analysis

To confirm the presence of specific minor splicing alterations, RNA-seq was performed on mononuclear blood cells from kindred 1 (the affected son, that is, patient #2, and the unaffected carrier father and younger male sibling) and from kindred 2 (the affected son, that is, patient #3, and the unaffected carrier father). Sample clustering by gene expression showed perfect separation between the two affected and three unaffected samples (Supplementary Fig. 4).

**Figure 4:**
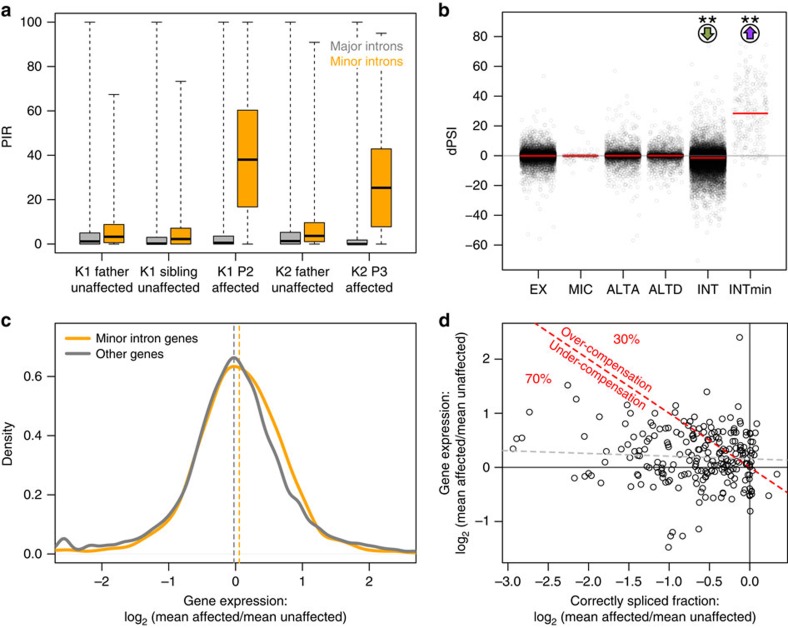
Summary of RNA-seq findings. (**a**) PIR for major (grey) and minor (orange) introns, for kindred 1 and kindred 2 subjects, showing specific minor intron retention in affected subjects compared with unaffected subjects. (**b**) Affected–unaffected average difference in percentage alternative splicing (dPSI) for different alternative splicing categories (ALTA, alternative 5′ splice site; ALTD, alternative 3′ splice site; EX, cassette exon; INT, major intron retention; INTmin: minor intron retention; MIC, micro-exon); significant deviation from 0 is observed only for major introns (small yet significant reduction in affected) and minor introns (significant increase in affected). (**c**) Log2 of the mean expression ratio between affected and unaffected, for minor intron genes and all other genes, displaying a slight shift of minor intron genes towards higher expression in affected subjects. (**d**) Scatterplot showing that increased expression in affected relative to unaffected subjects (*y* axis, log2 ratio of the mean expression in the two groups) is insufficient to compensate for the increased minor intron retention (*x* axis, log2 ratio of the mean correctly spliced fraction in the two groups), with 30% of the genes over-compensated and 70% of the genes under-compensated (separated by the red dashed line); the linear correlation between the expression ratio and correctly spliced ratio is negative but modest (grey dashed line).

We used a comprehensive set of curated splicing junctions and major/minor intron coordinates to evaluate splicing alterations, together with a recently developed RNA-seq analysis pipeline (vast-tools)^23^. We detected very consistent minor intron retention in affected subjects compared with unaffected subjects (median percentage of transcripts with intron retention, percentage intron retention (PIR) 25–40% for affected and PIR 2–4% for unaffected subjects, Wilcoxon two-tailed *P* value <10^−15^, Fig. 4a). These splicing changes were highly specific to minor introns, since major intron retention was small for all subjects (median PIR 0–1.5%, Fig. 4a). Moreover, other types of alternative splicing (that is, involving alternative cassette exon, microexons and 5′ or 3′ alternative splice sites) did not display significant difference between affected and unaffected subjects (Fig. 4b).

Interestingly, transcripts of minor intron genes displayed slightly increased (rather than reduced) expression levels in affected subjects, compared with other genes (edgeR^24^ median log2 expression ratio between affected and unaffected subjects: 0.054 for minor intron genes and −0.024 for other genes, Wilcoxon two-tailed *P* value=1.419e-05, Fig. 4c). That is expected to successfully compensate the increased minor intron retention only for a minority of the genes (30%, Fig. 4d). This suggests a compensatory transcriptional upregulation of minor intron genes, which however is not fully successful at restoring optimal levels of correctly spliced transcripts.

Analysis of gene expression and minor intron retention using other RNA-seq analysis methods (cufflinks^25^, DESeq^26^ and iReckon^27^) led to similar findings (Supplementary Tables 6–14, Supplementary Fig. 5 and Supplementary Note 5).

### Prioritization of genes altered in Roifman Syndrome

Minor intron genes are overall enriched in functions and phenotypes relevant to Roifman Syndrome (for example, brain and skeletal phenotypes, cell cycle regulation and signalling pathways, see Supplementary Fig. 6). To further prioritize genes whose splicing alteration leads to organ abnormalities observed for Roifman Syndrome, we integrated RNA-seq results based on different analytical methods (gene expression: edgeR, DESeq and cufflinks; splicing alteration detection: vast-tools, cufflinks and iReckon). We identified 83 genes for which at least two methods detected increased minor intron retention and decreased correctly spliced transcripts (Supplementary Data 4). These genes were annotated for human and mouse phenotypes to further prioritize genes whose minor intron retention is expected to be more relevant to Roifman Syndrome pathogenesis (Supplementary Data 5). We thus identified 30 genes with phenotypes relevant to Roifman Syndrome (developmental, skeletal, immune, neurocognitive and retinal); of these, the most compelling candidates were *ALG12*, *XRCC5* and *SMC3*. Their expression changes in Roifman Syndrome are summarized in Table 3, showing consensus detection of markedly increased minor intron retention and correctly spliced transcript isoform reduction; finally, for all three genes, minor intron retention is predicted to result into nonsense-mediated decay (Supplementary Data 4). *ALG12* is an alpha-1,6-mannosyltransferase implicated in a recessive glycosylation disorder^28^ (OMIM 607143) with a phenotypic presentation similar to Roifman Syndrome. *XRCC5* is a double-strand break repair gene also important for T-cell and B-cell receptor V(D)J recombination, whose mouse homozygous knockout causes growth retardation, severe combined immunodeficiency and retinal abnormalities^29^,^30^,^31^. *SMC3* is a component of the chromosome cohesion complex implicated in the dominant Cornelia de Lange syndrome 3 (OMIM 610759), which is characterized by hand and feet abnormalities, and in some instances also mild intellectual disability^32^.

We additionally investigated genes whose correctly spliced transcripts are almost completely abrogated, using very stringent thresholds. We thus identified three genes: *SLC9A9*, *WDFY1* and *ZCCHC8*. *SLC9A9* was implicated by one study in autism and epilepsy risk^33^, while *WDFY1* and *ZCCHC8* are not implicated in any human genetic disorder or mouse abnormal phenotypes. However, *ZCCHC8* is part of the NEXT (nuclear exosome targeting) complex, which is important for guiding the exosome degradation of malformed or byproduct transcripts such as PROMPTs (promoter upstream transcripts)^34^. Since reduced exosome function may contribute to increased persistence of minor intron retention transcripts, we investigated the splicing expression levels of the exosome core components and found two of them (*EXOSC1* and *EXOSC5*) among the 83 prioritized genes, even though they have not been implicated in human genetic disorder or mouse phenotype yet. Detailed expression changes for these genes are also reported in Table 3.

## Discussion

Using whole-genome sequencing of two affected siblings and targeted Sanger sequencing of four unrelated families, we have demonstrated that Roifman Syndrome is caused by compound heterozygous single-nucleotide variants (SNVs) in the minor spliceosomal snRNA gene *RNU4ATAC*, which was already implicated in a distinct and severe congenital disorder, MOPD1. Although both syndromes present growth and multi-system abnormalities, Roifman Syndrome has a different phenotype than MOPD1, even when considering the milder form of the latter. In addition, Roifman Syndrome causal variants always present a characteristic compound heterozygosity pattern: one variant affects *RNU4ATAC* elements already implicated in MOPD1, while the other variant affects a newly disease-implicated yet highly conserved element, the stem II.

RNA-seq analysis of two affected and three unaffected subjects revealed significantly higher minor intron retention in Roifman Syndrome patients compared with controls, which leads to reduced levels of correctly spliced transcripts for minor intron genes; we have also demonstrated that transcriptional alterations are highly specific of minor introns. Perhaps surprisingly, Roifman Syndrome patients did not exhibit overall reduced abundance of minor intron gene transcripts, as expected from a cell line model of short-term response to reduced minor spliceosome capacity^35^. On the contrary, a mild increase was detected, yet insufficient to fully compensate the splicing alterations. This finding is compatible with other reports showing that transcripts retaining minor introns can accumulate in the cell without being fully cleared by degradation machinery^36^,^37^; it may be further explained by partially reduced exosome functionality^38^, since two of its core components (*EXOSC1* and *EXOSC5*) and one component of the nuclear exosome targeting complex (*ZCCHC8*) have reduced correctly spliced transcripts. In addition, it is also possible that a feedback response drives increased transcription for incorrectly spliced minor intron genes.

While Roifman Syndrome and MOPD1 are extremely rare, recurrent spontaneous abortions or congenital disorders with a broader phenotypic spectrum may be caused by homozygous or compound heterozygous variants altering any of the *RNU4ATAC* structural elements critical for splicing, with an estimated prevalence up to 1 in 30,000 pregnancies (Supplementary Note 3). Since *RNU4ATAC* is not targeted by many commercially available exome capture kits (Supplementary Figs 7–9 and Supplementary Table 15), its contribution to Mendelian disorders may have been missed in other studies. This may also be the case for other noncoding minor spliceosome snRNA genes (*RNU6ATAC*, *RNU11* and *RNU12*), which, unlike their major spliceosome snRNA counterparts, are present at single loci in the genome and can thus act as recessive Mendelian disease genes.

## Methods

### Patient information

Patients or legal guardians provided informed consent in accordance with our Primary Immunodeficiency Registry & Tissue Bank protocol, Research Ethics Board Number 1000005598. Patients 1, 2, 3, 4 and 5 were consented in our facility; patients 1, 2 and 3 have included consent to publish photos. Patient 6 signed our institutional consent for genetic analysis as well as signing the Nature informed consent.

### Whole-genome sequencing of kindred 1

The whole genomes of the two affected siblings from kindred 1 were sequenced using the Complete Genomics platform^39^. The concentration of genomic DNA sample was measured by picogreen in triplicates and DNA quality was checked on a 2% agarose gel. About 11 μg of DNA was submitted to Complete Genomics for whole-genome sequencing. Complete Genomics employs high-density DNA nanoarrays that are concatamers of mate pair reads each ∼500-bp long. Base identification is performed using a proprietary non-sequential, unchained read technology known as combinatorial probe-anchor ligation. Each mate pair includes 35 nucleotides of genomic DNA sequence as well as adaptor sequences required for combinatorial probe-anchor ligation sequencing; the average mate gap length is 300 bp. For both subjects, >97% of the genome was covered at depth ≥5 by uniquely aligned reads (Supplementary Table 3).

The following variants were called by the Complete Genomics pipeline (version 2.0.2, human genome reference hg19) (ref. 40): (i) SNVs and small insertions/deletions (indels); (ii) structural variants (based on abnormal junction and discordant mate pair clusters, with size typically 50–75,000 bp); (iii) copy number variants (based on normalized sequencing coverage, with size typically >2,000 bp). Whole-genome variant data are available on request (please contact the corresponding authors).

### SNV and indel annotation and prioritization

Complete Genomics SNV and indels were annotated using a custom pipeline based on Annovar (August 2013 version)^41^, RefSeq gene models (downloaded from UCSC 2013 February 12), publicly available as well as internal databases for allele frequency (1000 Genomes^42^, NHLBI-ESP^43^ and internal Complete Genomics control databases), genomic conservation (UCSC PhyloP and phastCons for placental mammals and 100 vertebrates^44^) and variant impact predictors (SIFT^45^, PolyPhen2 (ref. 46), Mutation Assessor^47^ and CADD^48^). Please see the Supplementary Note 6 for a detailed description of the annotation fields and database versions.

Annotated variants mapping to coding or noncoding exonic sequence were further prioritized according to these criteria: (i) sequencing quality; (ii) allele frequency (restricting to rare variants); (iii) conservation and predicted impact (restricting to variants potentially damaging the gene product); (iv) variant pathogenic effect, as reported by disease variant databases; (v) zygosity and genic mode of inheritance; and (vi) human disease and mouse abnormal phenotypes in which a gene is known to be implicated. Only high-quality, rare and potentially damaging variants were prioritized; while homozygous and potential compound heterozygous variants were reported also for genes not implicated in human genetic disorders, heterozygous variants were reported only for genes implicated in a known dominant disorder according to Human Phenotype Ontology^49^ or Clinical Genomics Database^50^. A detailed description of the prioritization rules can be found in the Supplementary Note 7.

### Copy number variant (CNV) annotation

Copy number gains and losses, reported by the Complete Genomics pipeline in the ‘cnvSegmentsDiploidBeta' file, were separately annotated for frequency based on 50% reciprocal overlap with CNVs called in 54 unrelated control samples from the Complete Genomics diversity panel (pipeline version 2.0), and overlap with CNVs from the Database of Genomic Variants (November 2010 and March 2013 versions)^51^; CNVs were also annotated for overlapping gene transcripts and exons (RefSeq, downloaded March 2013). CNVs overlapping at least one genic exon and not found in the Complete Genomics diversity panel were inspected manually.

### Minor intron identification

The U12db (ref. 52) was previously used as the authoritative source for minor introns^10^,^11^,^12^. However, it was last updated in January 2007 and it is based on the outdated genome build hg17/NCBI35. For this reason, we preferred to use U12db major and minor intron sequences to extract splicing consensus sequences, construct position-specific score matrices (PSSMs)^53^, and re-classify up-to-date hg19 RefSeq introns.

The intron 5′ initial 15 bp (including the 5′ recognition splicing consensus sequence) and the intron 3′ terminal 39 bp (including the branching site splicing consensus sequence) were downloaded from U12 db for 487 GTAG minor introns, 208 ATAC minor introns and 82 major introns. The initial and terminal intronic dinucleotides, corresponding to the highly conserved GT/AT and AG/AC sequences, were removed, as they are not highly discriminant of major and minor introns. The resulting sequences were merged into 50-bp-long sequences, and MEME 4.9.1 (ref. 54) was used to identify over-represented sequences and construct corresponding position probability matrix. As expected, for minor introns we identified two over-represented sequences, corresponding to the 5′ recognition consensus (ATCCTT, followed by less-conserved bases) and the branching site (TTTCCTT[A/G]AC, surrounded by less-conserved bases); for major introns, we identified only the 5′ recognition consensus sequence (AAGTTT), while the branching site consensus is too degenerate and no over-represented sequence was found. These consensus sequences had good correspondence to curated ones^9^.

We next created log-odd (LOD) PSSM by dividing each nucleotide probability by 0.25; while this is based on the simplistic assumption that the appearance of A, C, G or T is equally likely, we use the LODs to discriminate intronic sequences with high score either for the minor or for the major consensus sequences, thus the assumption is acceptable. In case the frequency of the base was 0, we assigned the LOD score of −100.

We scanned the 5′ and 3′ sequences of known introns (hg19 RefSeq, downloaded from UCSC in April 2014) and calculated the following PSSM match scores (representing the LOD-transformed probability of observing the intronic sequence based on the LOD PSSM matrix): (a) for each intronic 5′ sequence we calculated the minor intron 5′ recognition match score and the major intron 5′ recognition match score; (b) for each intronic 3′ sequence we calculated the minor intron-branching site match score. In particular, for the intron 5′ we calculated the match score based on the 13 bases after the initial conserved dinucleotide, as in the MEME analysis we always observed the over-represented consensus sequence at that position; for the intron 3′, we slided a 19-base window over the last 40 bases (excluding the terminal conserved dinucleotide), calculated the match score for each window, and selected the maximum match score, as the MEME analysis showed a variable position of the corresponding over-represented sequence in the 3′ sequence. The match score represents the LOD-transformed probability of observing the intronic sequence based on the LOD PSSM matrix, and was defined as:


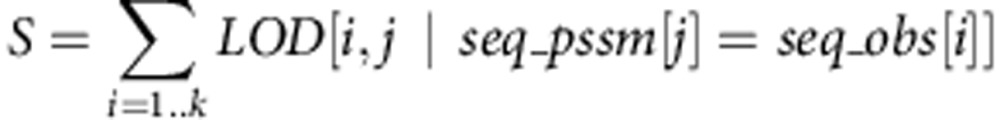


where *i* represents the *i*-th position in the scanned sequence, and *LOD*[*i*, *j|seq_pssm*[*j*]=*seq_obs*[*i*]] represents the LOD score value at the *i*-th position of the PSSM for the nucleotide observed at position *i* in the scanned sequence; this notation is based on PSSM with columns (*j* index) corresponding to the four nucleotides (*seq_pssm*={A, C, T, G}) and PSSM rows (*i* index) corresponding to specific positions.

Comparing the putative minor introns found in this analysis with the minor introns reported in U12db, we found that requiring a score <2 for the 5′ match to the 5′ minor intron recognition PSSM and for the 3′ match to the minor intron-branching site PSSM was effective at discriminating minor introns from major introns. This lead to identification of 822 unique introns and 744 unique minor intron genes.

### RNA-seq extraction and sequencing

RNA-seq was performed on mononuclear blood cells from three members of kindred 1 (affected son, corresponding to patient #2, unaffected father and sibling) and two members of kindred 2 (affected son, corresponding to patient #3 and unaffected father).

Total RNA sample quality control was performed using an Agilent Bioanalyzer 2100 RNA Nano chip and following the Agilent Technologies' recommendation. RNA library preparation was performed following the Illumina TruSeq RNA Sample Preparation V2 Guide (Rev. D, September 2012). Briefly, 1 μg of total RNA was used as the input material; poly(A) mRNA were enriched with oligo dT beads and the enriched fraction was fragmented for 6 min at 94 °C; fragmented RNA was converted to double-stranded cDNA; end-repaired and adenylated at the 3′ to create an overhang A to allow for ligation of TruSeq adapters with an overhang T; library fragments were then amplified under the following conditions: initial denaturation at 98 °C for 10 s, followed by 14 cycles of 98 °C for 10 s, 60 °C for 30 s and 72 °C for 30 s, and finally an extension step for 5 min at 72 °C; at the amplification step, each sample were amplified with a different barcoded adapters to allow for multiplex sequencing. A volume of 1 μl of the final RNA libraries was loaded on a Bioanalyzer 2100 DNA High Sensitivity chip (Agilent Technologies) to check for size; RNA libraries were quantified by quantitative PCR using the Kapa Library Quantification Illumina/ABI Prism Kit protocol (KAPA Biosystems). Libraries were pooled in equimolar quantities and paired-end sequenced on an Illumina HiSeq 2500 platform using a Rapid Run Mode flowcell and the V3 sequencing chemistry following Illumina's recommended protocol to generate paired-end reads of 100 bases in length.

### RNA-seq bioinfomatics pre-processing and quality control (QC)

Reads were trimmed to remove adapters and low-quality ends using Trimgalore v0.3.3 ( http://www.bioinformatics.babraham.ac.uk/projects/trim_galore/), resulting in 27,971,870–38,088,242 paired-end reads; additional QC checks were performed using FastQC v0.11.2 ( http://www.bioinformatics.babraham.ac.uk/projects/fastqc/). The human rRNA content (3.74–12.8% of the trimmed reads) was assessed using FastQ Screen ( http://www.bioinformatics.babraham.ac.uk/projects/fastq_screen/); 5.8S rRNA, 5S rRNA (variants 1–17), 18S rRNA and 28S rRNA human sequences were retrieved from RefSeq (June 2014). Alignment and QC statistics are summarized in Supplementary Table 16.

### RNA-seq bioinfomatics alternative splicing analysis

Alternative splicing was analysed using *vast-tools* (version 0.2.1), a multi-module computational pipeline^23^,^55^, publicly available at https://github.com/vastgroup/vast-tools. This pipeline uses a broad range of evidence sources (RNA-seq, EST and cDNA, gene annotations and evolutionary conservation) to define splice junctions for the human transcriptome (hg19). That results in 258,603 potential alternative splicing events, comprising 74,233 cassette exon events, 478 microexons, 12,677 alternative 5′-splice sites, 18,094 alternative 3′-splice site and 153,131 introns (including 666 minor introns). Of note, introns were considered even if they have never previously been detected as retained.

Read-1 and read-2 from RNA-seq paired-end reads were separately processed to produce two pairs of 50-bp fragments; this step is required for optimal mapping to junctions and counting. Whenever presence of adaptor sequences or low-quality stretches produce trimmed read lengths below 100 bp, the two 50-bp fragment pairs can overlap partially. Fragments mapping to multiple sites in the human genome, or to sites with overlapping transcribed sequence belonging to different genes, were discarded. Remaining fragments were aligned to libraries of exon–exon and exon–intron junction sequences, using bowtie with settings −m 1 −v 2; the 50-bp fragment pairs were mapped independently, while tracking from which original 100-bp read pair they were derived. We then counted how many original 100-bp read pairs were represented at each junction, and counts were finally normalized by the number of uniquely mappable 50-mer positions in each junction sequence. Per cent-spliced-in or PIR scores were calculated as previously described^23^,^55^,^56^,^57^.

Raw output from this pipeline was filtered using associated quality information. For cassette exons, we required a coverage score of ‘SOK', ‘OK' or ‘LOW' (roughly corresponding to a minimum number of mapped reads per junction of 100, 20 or 15, respectively), and a junction balance score of ‘OK' or ‘B1' (corresponding to a ratio of numbers of reads mapping to the upstream and downstream junctions of less than twofold, or between two- and fivefolds, respectively; for details, see https://github.com/vastgroup/vast-tools). For alternative 5′- and 3′-splice sites and microexons, we required a coverage score of ‘SOK', ‘OK' or ‘LOW'. For intron retention events, we required a coverage of ≥15 total reads per event and a junction balance binomial test *P* value >0.05.

For all alternative splicing events, only events detected in at least two individuals were retained for analyses comparing affected and unaffected individuals. Differential PSI/PIR between affected and unaffected individuals were calculated as differences between averages in these groups.

Fold differences in correctly spliced fraction with respect to minor intron retention events (Fig. 4d) were calculated as log2 ((100−PIR_affected_)/(100−PIR_unaffected_)).

Predictions of introns whose retention triggers nonsense-mediated decay were derived from a previous publication^23^.

The R package edgeR (ref. 24) was used to assess differential expression for the over/under-compensation assessment, please see next section for more details.

Note that, for different RNA-seq methods, the term ‘fold change' means the ratio between the expression level (or other quantitative measure) between two conditions (affected/unaffected whenever not explicitly defined).

### RNA-seq bioinfomatics gene and isoform expression

Trimmed reads were aligned to the human reference sequence (hg19) using TopHat v2.0.10 (ref. 58) (82.6–91.5% of the trimmed reads were concordantly aligned). For the edgeR and DESeq differential gene expression analysis, read counts for genic exonic sequences were extracted from TopHat alignments using HTseq v2.6.4 (ref. 59), with the ‘intersection strict' setting (‘intersection strict' ensures that reads only partially overlapping exons are not counted).

For the edgeR (ref. 24) analysis, library size normalization factors were calculated using the method ‘trimmed mean of M-values (TMM)' and differential analysis was perfomed using the generalized linear models functions with default settings, with a design matrix specifying phenotype (affected and unaffected); at a false discovery rate (FDR) <0.05, there were 500 differentially expressed genes.

For the DESeq differential intron expression, intron coordinates and corresponding gene symbols were based on RefSeq (downloaded from UCSC, April 2014); for every gene, overlapping introns were merged and the parts overlapping exons were removed; finally, intron read counts were extracted from TopHat alignments using HTseq v2.6.4. For both analyses, DESeq v1.16.0 (ref. 26) was used for normalization and for testing differential expression.

Cufflinks/cuffmerge v2.0.2 (ref. 25) were used to assemble transcript isoforms from aligned reads, and cuffdiff to test for differential gene expression, differential transcript isoform expression and differential splicing output; the cufflinks pre-mRNA-fraction and min-isoform-fraction parameters were relaxed to 0.05 to maximize sensitivity. iReckon v1.0.8 (ref. 27) was also used to assemble transcripts from aligned reads and assess intron retention.

To confirm segregation of case and control samples, clustering was performed using the R package CummeRbund v2.6.1, based on the fragments per kilobase of exon per million mapped fragments (FPKM) estimates generated by cufflinks.

### Prioritization of genes altered in Roifman Syndrome

For the genes with minor introns (744 genes), we integrated results from different tools into a final score, indicating how many methods supported the presence of minor intron splicing retention and reduced correctly spliced transcript.

For DESeq, edgeR and cufflinks differential gene expression analysis, we imported the log2 affected/unaffected fold change, the nominal *P* value and the FDR *q* value; for DESeq and cufflinks, we also imported the mean normalized counts (DESeq) and FPKM (cufflinks) for the two conditions (affected and unaffected). We found DESeq and edgeR to be highly correlated (Spearman rho of log2 fold change: 0.993; Spearman rho of differential expression *P* value: 0.930), although edgeR found more genes significant for differential expression (at FDR≤10%, edgeR: 54 minor intron genes, DESeq: 12); DESeq and edgeR were also correlated to cufflinks (Spearman rho of log2 fold change: 0.916 and 0.907, respectively; Spearman rho of nominal *P* value: 0.697 and 0.581, respectively).

Vast-tools splicing results were restricted to minor intron retention events, and whenever a gene had more than one minor intron (48/666 minor intron genes in the vast-tools splicing analysis), we imported only the one with the largest difference between affected and unaffected minor intron PIR. We used the minor intron PIR to calculate the log2 affected/unaffected fold change in correctly spliced transcript as: log2 ((100−PIR_RS)/(100−PIR_CT)). We derived the percentage of genes with over- or under-compensation in Fig. 4d by comparing the log2 affected/unaffected fold change in expression from edgeR (*y*, following Fig. 4d) to the log2 affected/unaffected fold change in correctly spliced transcript from vast-tools (*x*, following Fig. 4d): when *y*+*x*<0, there is under-compensation (that is, net decrease in correctly spliced transcript); percentages were reported with respect to minor intron genes without missing values for vast-tools and edgeR (222/744 genes). For the combination of edgeR and vast-tools, we considered genes having a minor intron splicing alteration when log2 (vast-tools fold change correctly spliced)+log2 (edgeR expression fold change) <log2 (1/1.2).

For cufflinks transcript isoform analysis, we defined the ‘correctly spliced isoform' as the isoform with highest expression level (FPKM) in unaffected, and the ‘minor intron retention isoform' as the one with minor intron retention and highest expression level (FPKM) in affected subjects. Following these definitions, we defined genes having a minor intron splicing alteration supported by cufflinks when they met the following requirement: ‘correctly spliced isoform' log2 affected/unaffected FPKM fold change <log2 (1/1.2) and ‘minor intron retention isoform' log2 affected/unaffected FPKM fold change >log2 (1.2).

For iReckon transcript isoform analysis, we defined ‘correctly spliced' the isoforms recognized by iReckon as ‘known', and ‘minor intron retention' the isoforms recognized by iReckon as ‘intron retention' and including a minor intron; we then calculated total expression levels (reads per kilobase of exon per million reads mapped (RPKM)) for the two isoform groups, and we considered genes having a minor intron splicing alteration when they met the following requirement: ‘correctly spliced isoform' log2 affected/unaffected RPKM fold change <log2 (1/1.2) and ‘minor intron retention isoform' log2 affected/unaffected RPKM fold change >log2 (1.2).

We found a significant final score agreement between vast-tools+edgeR and cufflinks (two-tailed Fisher's exact test *P* value=3.033e-08 and odds ratio=8.73), and between cufflinks and iReckon (two-tailed Fisher's exact test *P* value=6.788e-09 and odds ratio=4.87), while only the agreement between vast-tools+edgeR and iReckon was more modest (two-tailed Fisher's exact test *P* value=0.2036 and odds ratio=1.57), overall suggesting that this procedure is adequate to integrate results from different methods.

Genes whose correctly spliced transcripts are almost completely abrogated were defined as having vast-tools minor intron PIR_RS >85% and PIR_CT <30% and at least two of these three conditions met: cufflinks correctly spliced isoform log2 affected/unaffected FPKM fold change <−2, iReckon correctly spliced isoform log2 affected/unaffected FPKM fold change <−2, log2 (vast-tools fold change correctly spliced)+log2 (edgeR expression fold change) <−2.

## Additional information

**How to cite this article:** Merico, D. *et al.* Compound heterozygous mutations in the noncoding *RNU4ATAC* cause Roifman Syndrome by disrupting minor intron splicing. *Nat. Commun.* 6:8718 doi: 10.1038/ncomms9718 (2015).

## Supplementary Material

Supplementary Information Supplementary Figures 1-9, Supplementary Tables 1-17, Supplementary Notes 1-7 and Supplementary References.

Supplementary Data 1 Whole genome sequencing of kindred 1, prioritized SNV and indel.

Supplementary Data 2 Whole genome sequencing of kindred 1, annotated and prioritized copy number variants (overlapping at least one genic exon and not found in the Complete genomics diversity panel).

Supplementary Data 3 Whole genome sequencing of kindred 1, annotated and prioritized structural variants (overlapping at least one gene and not found in the Complete genomics diversity panel).

Supplementary Data 4 RNA-seq gene expression and splicing statistics for minor intron genes (based on cufflinks, DESeq, edgeR, iReckon, vast-tools).

Supplementary Data 5 Phenotype and functional annotations for minor intron genes with at least two out of three RNAseq methods (edgeR + vast-tools, cufflinks, iReckon) detecting minor intron retention and decreased expression of correctly spliced isoforms.

## Figures and Tables

**Table 1:** Roifman Syndrome compound heterozygous variants detected in six affected individuals.

**Subject, kindred**	**Allele**	**U4atac snRNA pos**	**Sub**	**Freq 1000G**	**Freq cg1KG**	**Freq cgW597**	**PhyloP PMam**	**MOPD1/Novel**	**dbSNP**
1–2 K1	Pat	13	C>T	0	0	0.0008	2.57	Novel	—
1–2 K1	Mat	37	G>A	0	0	0	2.63	Novel	—
3, K2	Pat	13	C>T	0	0	0.0008	2.57	Novel	—
3, K2	Mat	48	G>A	0	0	0	2.63	Novel	—
4–5, K3	Mat	16	G>A	0	0	0.0008	2.63	Novel	—
4–5, K3	Pat	51	G>A	0.0014	0	0	1.37	MOPD1	rs188343279
6, K4	Mat	8	C>T	0	0.0011	0	2.57	Novel	rs370715569
6, K4	Pat	118	T>C	0	0	0	2.12	Novel	—

Subject, kindred, subject and kindred index; Allele, maternal or paternal allele indication (all variants are coumpound heterozygous); U4atac snRNA pos, U4atac snRNA position (1 corresponds to the genomic coordinate 122,288,456 on chromosome 2, hg19 reference); Sub, substitution (reference>alternate); Freq 1000G, Freq cg1kG, Freq cgW597, alternate allele frequency in the 1000 Genomes project, and in the internal Complete Genomics control databases based on the 1000 Genomes subset and the Wellderly study (436 and 597 subjects, respectively); PhyloP PMam, UCSC placental mammal PhyloP score of genomic nucleotide conservation (score>0 corresponds to negative selection); MOPD1/Novel, variant previously reported as causal for MOPD1, or reported for the first time as causing a genetic disorder; dbSNP, matching dbSNP138 record.

**Table 2:** Roifman Syndrome and MOPD1 phenotypic manifestations.

**Phenotypic feature**	**MOPD1**	**Roifman Syndrome**
Pre- and post-natal lethality	Yes	No
Absent or sparse hair	Yes	No
Dysplastic nails	Yes	No
Severe micrognathia	Yes	No
Agenesis of ear lobes	Yes	No
Philtrum	Variable	Long
Thin upper lips	No	Yes
Nose	Large/prominent, downturned nasal tip	Tubular and upturned
Proptotic eyes	Yes	No
Retinal changes	Hypopigmentation	Severe degradation of rods and cones
Skeletal abnormalities	Metaphyseal changes, flat acetabular root	Spondyloepiphyseal dysplasia
Immunodeficiency	No	Yes
Noncompaction of the heart	No	Yes
Head	Severe prenatal microcephaly	Mild microcephaly or normal head size
Structural brain abnormalities	Agenesis of corpus callosum, gyral anomalies, cortical atrophy, enlarged lateral ventricles, hypoplastic frontal lobes, hypoplastic pituitary gland, interhemispheric cysts, cerebellar vermis hypoplasia	None in all cases but in 1, who had partial agenesis of corpus callosum and hippocampal atrophy^6^
Intractable epilepsy	Yes	No
Endocrine dysfunction	Growth hormone deficiency, low prolactin levels	None

**Table 3:** Prioritized genes whose splicing alteration contributes to Roifman Syndrome.

**Gene**	**Expr. FC**	**VT Aff. MI PIR**	**VT Unaff. MI PIR**	**Cuff. MI FC**	**iReck. MI FC**	**VT CS FC**	**Cuff. CS FC**	**iReck. CS FC**	**Phen./Funct.**
*ALG12*	0.85–0.85	38.5%	8.0%	4.18	NA	0.57	0.62	0.56	Growth, neurodev., immune
*XRCC5*	1.07–1.18	28.2%	1.2%	Inf	7.95	0.79	0.99	0.57	Neurodev., immune, retinal
*SMC3*	0.92–1.17	31.9%	2.1%	15.25	15.57	0.64	0.85	0.48	Skeletal
*SLC9A9*	2.01–3.00	85.4%	2.7%	Inf	NA	0.31	0.00	0.00	Neurodev.
*WDFY1*	1.26–1.75	88.6%	12.8%	10.07	10.42	0.17	0.07	0.13	No phen., endosome
*ZCCHC8*	1.60–1.88	100%	27.1%	15.82	20.76	0.00	0.12	0.30	No phen., exosome NEXT
*EXOSC1*	0.87–0.93	NA	5.7%	Inf	Inf	NA	0.78	0.40	No phen., exosome core
*EXOSC5*	0.67–0.71	22.1%	9.1%	3.37	NA	0.57	0.59	0.41	No phen., exosome core

Cuff. CS FC, cufflinks correctly spliced isoform affected/unaffected fold change; iReck. CS, FC, iReckon correctly spliced isoform affected/unaffected fold change; Cuff. MI FC, cufflinks minor intron retention isoform affected/unaffected fold change; Expr. FC, overall expression affected/unaffected fold change (interval based on edgeR, DESeq and cufflinks estimates); Gene, official gene symbol; iReck. MI FC, iReckon minor intron retention isoform affected/unaffected fold change; Phen./Funct., gene phenotype and function (note that function is reported only in absence of phenotype information, that is, ‘No phen.'); VT aff. MI PIR, vast-tools minor intron retention percentage in affected; VT CS FC, vast-tools+edgeR correctly spliced isoform affected/unaffected fold change; VT unaff. MI PIR, vast-tools minor intron retention percentage in unaffected.
