# Supplementary material for: Compound heterozygous mutations in the noncoding RNU4ATAC cause Roifman Syndrome by disrupting minor intron splicing
Source: Nat Commun. 2015 Nov 2;6:8718. doi: 10.1038/ncomms9718 (PMC4667643; doi:10.1038/ncomms9718)
Supplement: Supplementary Information — Supplementary Figures 1-9, Supplementary Tables 1-17, Supplementary Notes 1-7 and Supplementary References. [file ncomms9718-s1.pdf]

SUPPLEMENTARY FIGURES

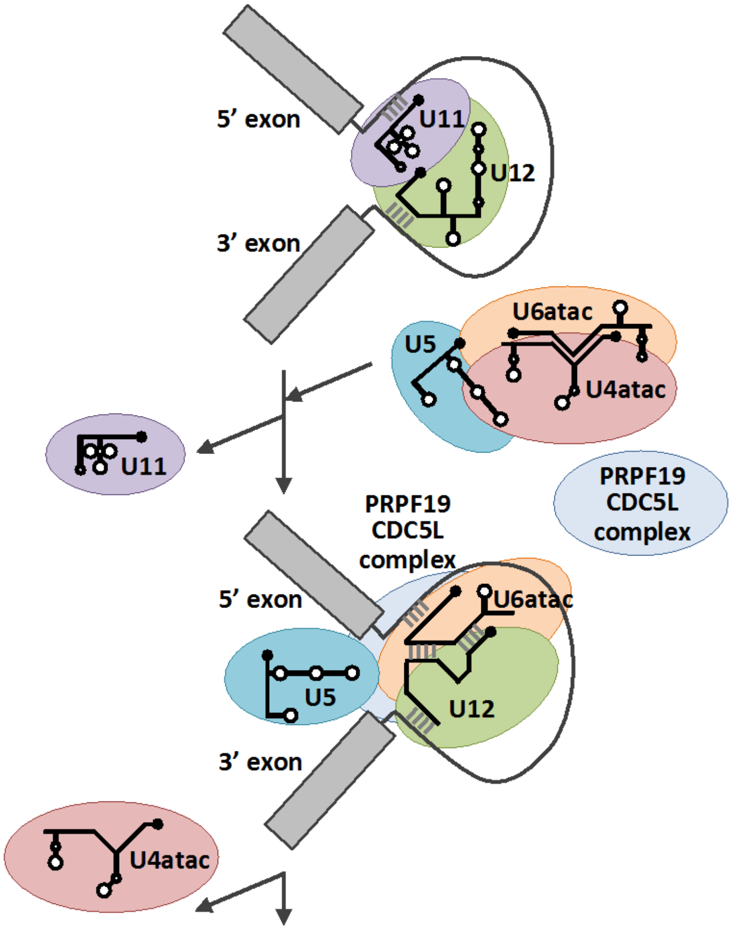

Supplementary Figure 1. Schematic depiction of the spliceosomal snRNPs and the non-snRNP PRPF19:CDC5L protein complex at various assembly stages. The PRPF19:CDC5L complex association with the U12-dependent spliceosome is inferred from the major spliceosome (figure adapted from [1]).

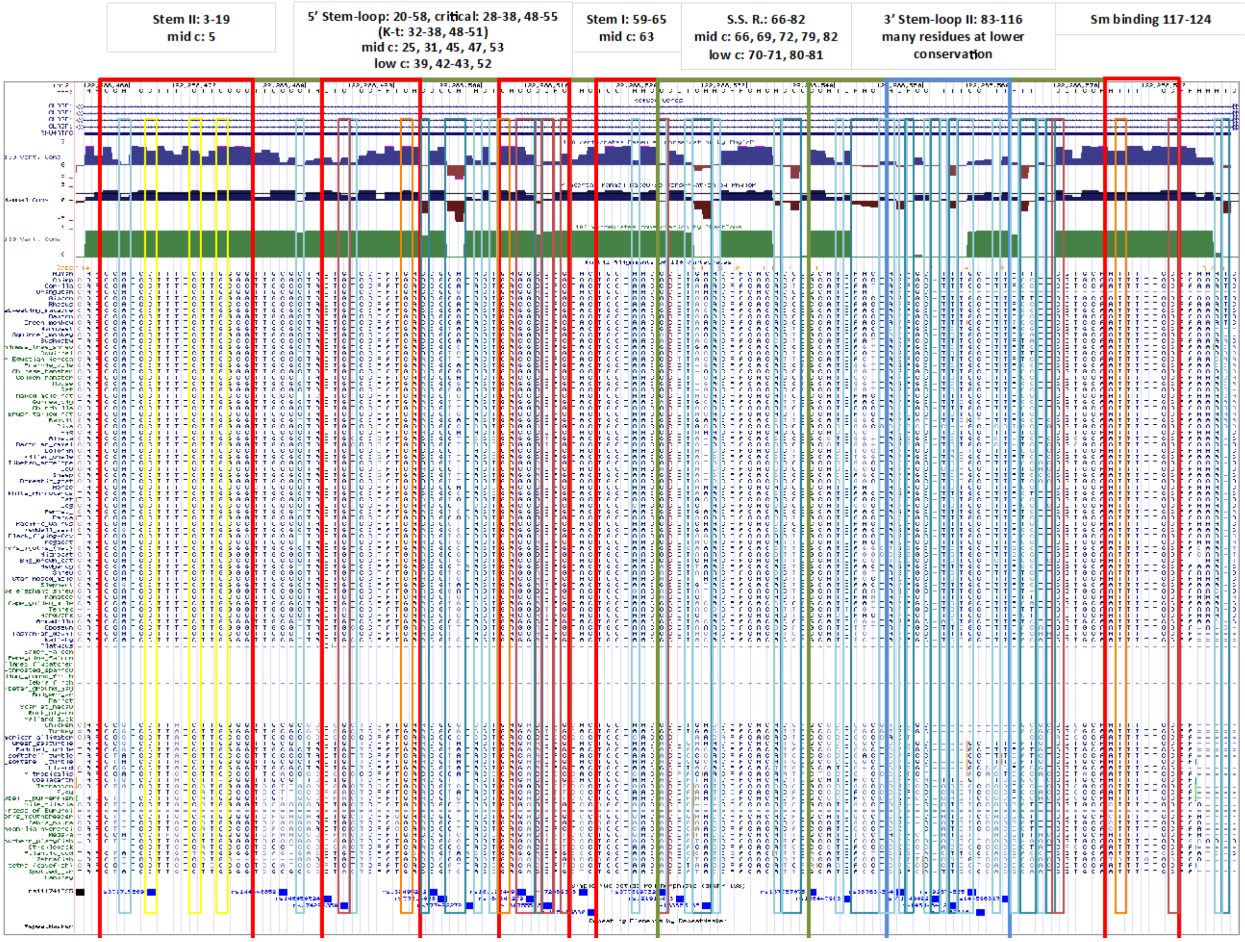

Supplementary Figure 2. *RNU4ATAC* conservation profile.

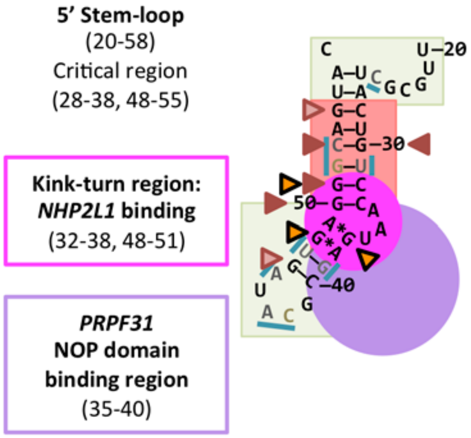

Supplementary Figure 3. *RNU4ATAC* 5' stem loop protein binding elements.

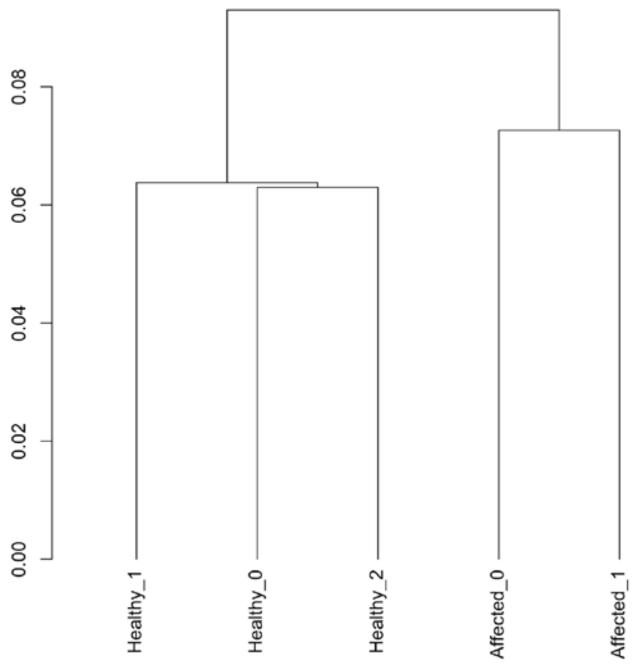

Supplementary Figure 4. RNA-seq sample clustering based on gene expression, showing separation between affected and unaffected.

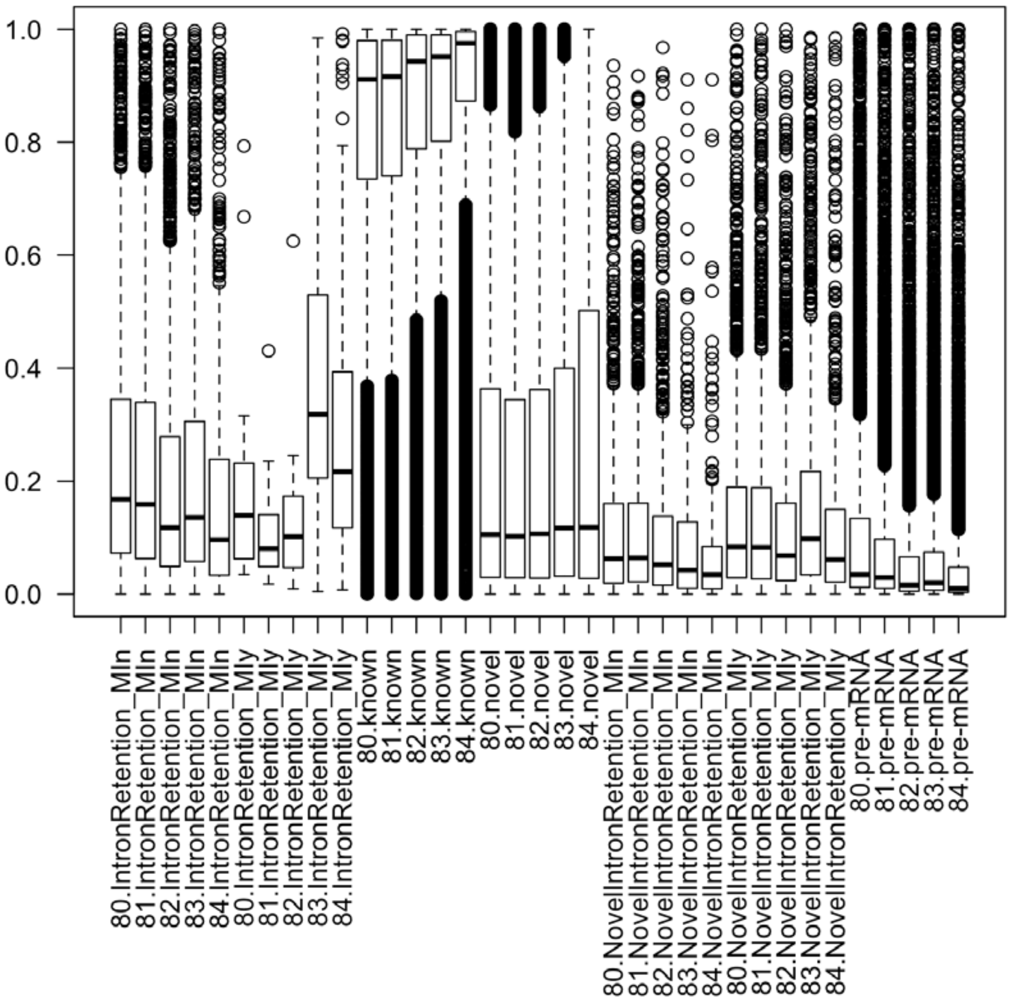

Supplementary Figure 5. Distribution of gene RPKM fraction, for different isoform categories and subjects (the RPKM fraction is calculated with respect to each isoform category, and adds up to 1 for each gene; if no isoform is detected within a given category, the fractions value is set to NA). Subject 80, 84: unaffected father and affected son from kindred 2; subject 81, 82, 83: unaffected father, unaffected son and affected son from kindred 1. The affected subjects (83 and 84) clearly display an increase in minor intron retention (IntronRetention_MIy) but not in major intron retention (IntronRetention_MIn).

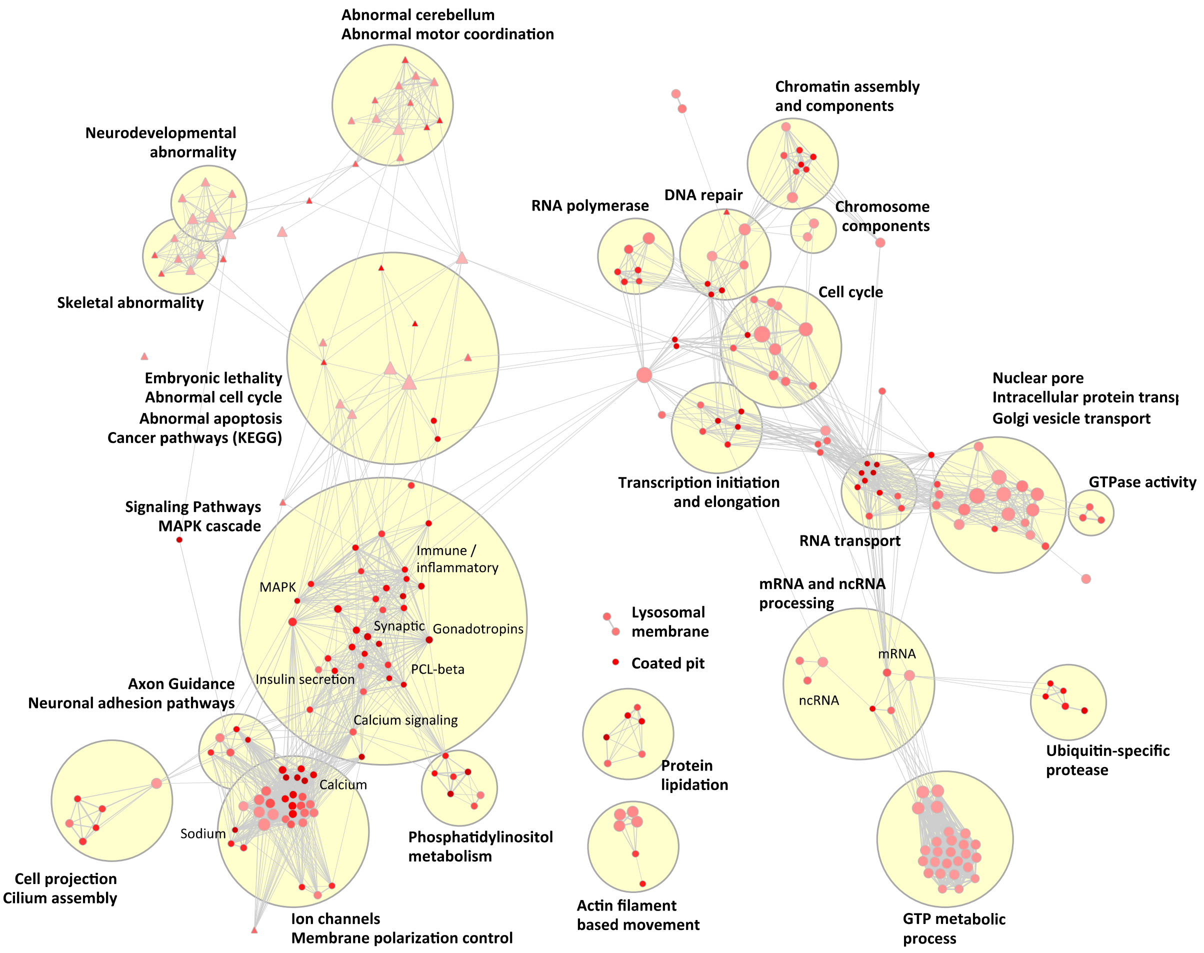

Supplementary Figure 6. Enrichment map of minor intron genes. We tested all minor intron genes for over-representation in functional gene-sets (Gene Ontology [2]: extracted from the R/Bioconductor package org.Hs.eg.db 2.14.0, KEGG [3]: downloaded from website May 2014, Reactome [4]: downloaded from website May 2014, Biocarta: downloaded from website May 2014, NCI-PID [5]: downloaded from website Sept 2013) and MPO mouse phenotypes from MGI (downloaded September 2014, http://www.informatics.jax.org/), using the Fisher's Exact Test. Enrichment results were visualized in Cytoscape v2.8.2 [6] using the plugin Enrichment Map v1.2 [7]. Gene-sets corresponding to abnormal mouse phenotypes are represented as triangles, while gene-sets corresponding to Gene Ontology terms or pathways are represented as circles; the triangle or circle color is proportional to enrichment; edges (connections) represent the degree of overlap among gene-sets; the weighted spring embedded layout was used to refine the layout, and gene-set clusters were manually identified and labeled (light yellow shades). As expected based on the phenotypic presentation of MOPD1 and Roifman Syndrome, we found over-representation in (a) functions and pathways related to development, cell cycle, DNA replication and repair, transcriptional regulation and RNA processing, ion channels, immunity, as well as (b) phenotypes related to neurodevelopment, skeleton and embryonic development.

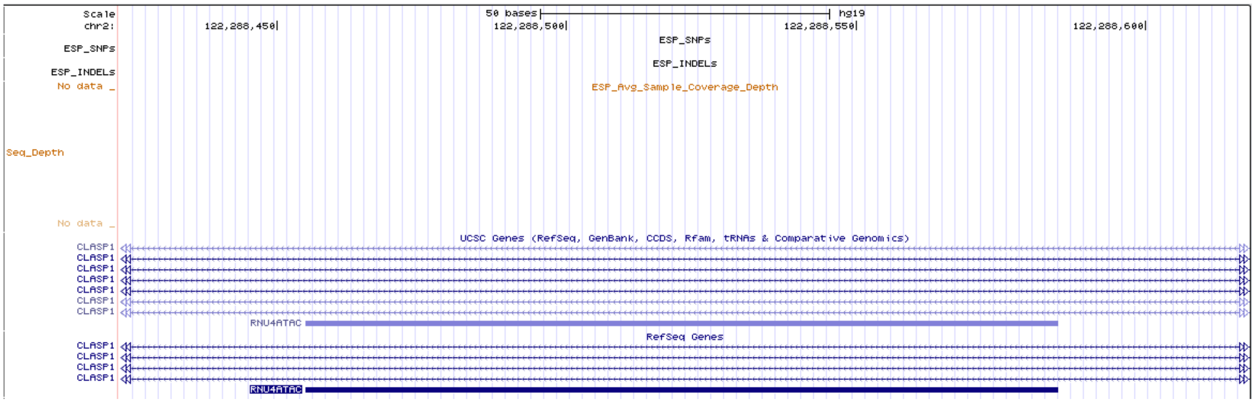

Supplementary Figure 7. Average sequencing depth of *RNU4ATAC* in the NHLBI-ESP [8] exomes, showing no coverage.

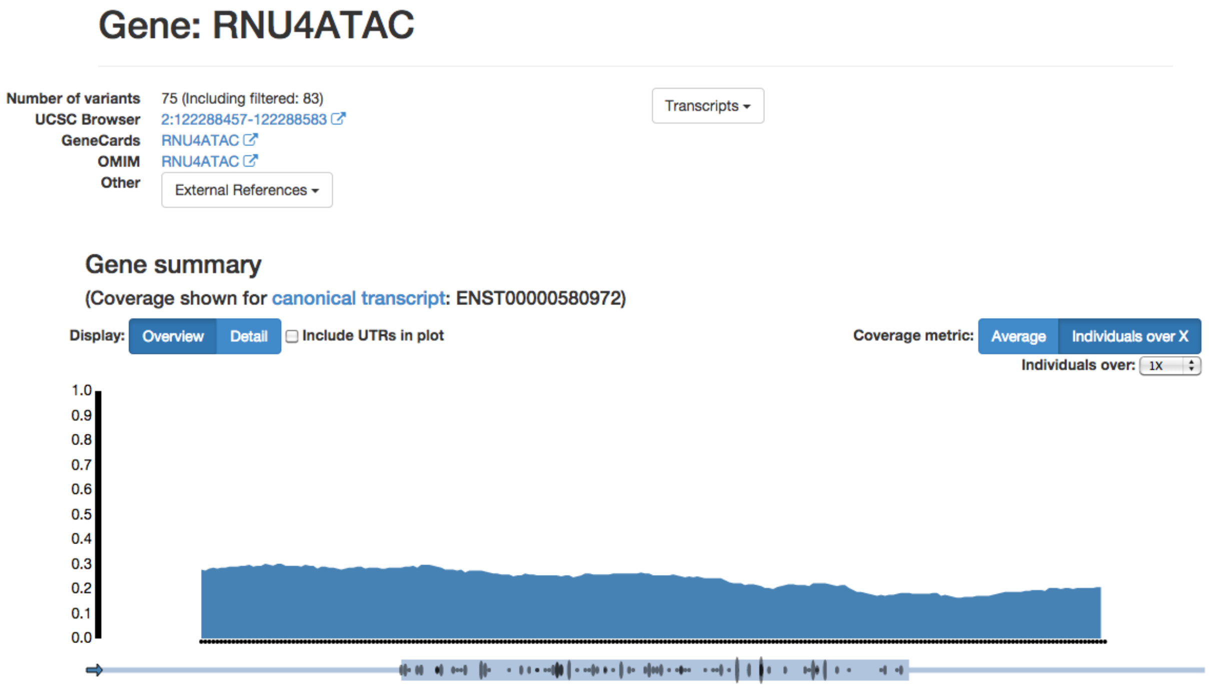

Supplementary Figure 8. Fraction of ExAC exomes with *RNAU4ATAC* sequencing depth > 1x, showing very poor coverage.

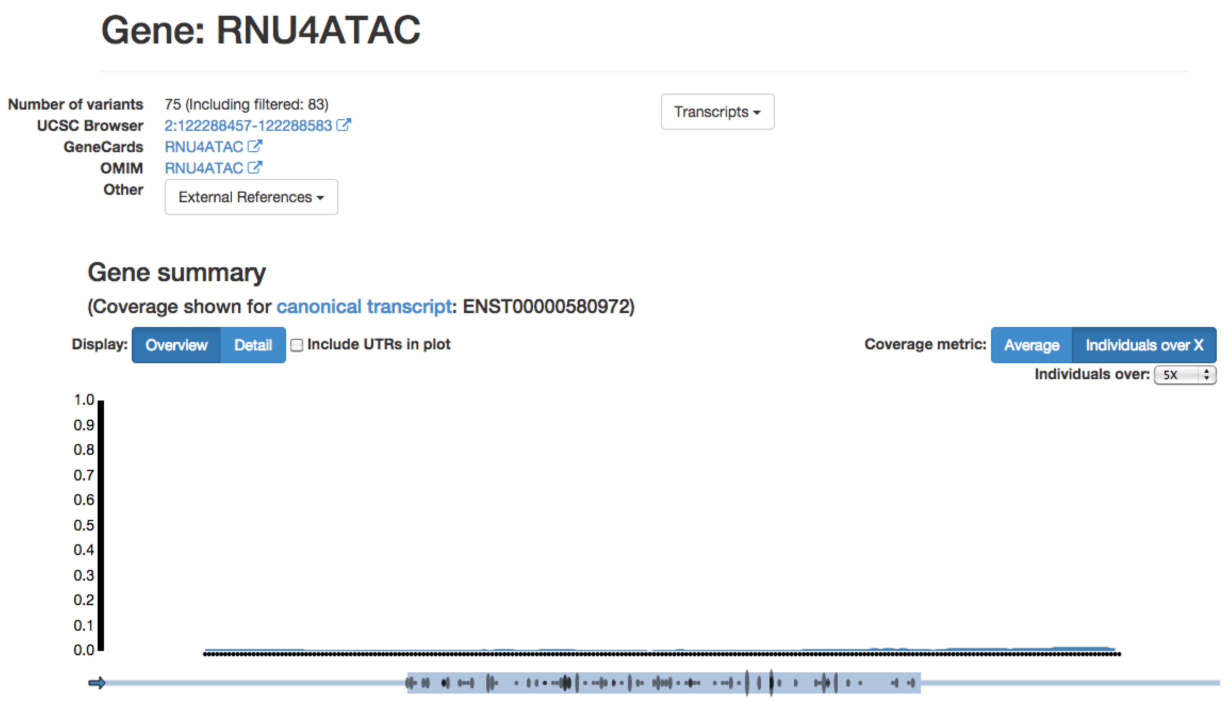

Supplementary Figure 9. Fraction of ExAC exomes with *RNAU4ATAC* sequencing depth > 5x, showing very poor coverage.

SUPPLEMENTARY TABLES

	Patients						
**Manifestations**	**1**	**2**	**3**	**4**	**5**	**6**	**Total**
**Sex**	M	M	M	F	M	M	5M, 1F
**Growth Deficiency**							
**- prenatal**	Yes	Yes	Yes	Yes	Yes	Yes	6/6
**- postnatal**	Yes	Yes	Yes	Yes	Yes	Yes	6/6
**Performance**							
**- cognitive delay**	Yes	Yes	Yes	Yes	Yes	No	5/6
**- hypotonia**	Yes	Yes	Yes	Yes	Yes	No	5/6
**Dysmorphic Features**							
**- microcephaly**	Yes	No	Yes	Yes	Yes	Yes	5/6
**- long philtrum**	Yes	Yes	Yes	Yes	Yes	Yes	6/6
**- thin upper lip**	Yes	Yes	Yes	Yes	Yes	Yes	6/6
**- narrow upturned nose**	Yes	Yes	Yes	Yes	Yes	Yes	6/6
**- 5th digit clinodactyly**	Yes	Yes	Yes	No	Yes	No	4/6
**- transverse palmar crease**	Yes	Yes	Yes	Yes	No	No	5/6
**- brachydactyly**	Yes	Yes	Yes	Yes	Yes	Yes	6/6
**Skeleton**							
**- epiphyseal dysplasia**	Yes	Yes	Yes	Yes	Yes	Yes	6/6
**- vertebral changes**	Yes	Yes	Yes	No	No	No	3/6
**- short metacarpals**	Yes	Yes	Yes	Yes	Yes	Yes	6/6
**Eye**							
**- retinal distrophy**	No	Yes	Yes	No	Yes	No	3/6
**Heart**							
**- non-compaction**	Yes	No	No	No	No	No	1/6
**- VSD**	No	No	No	Yes	No	No	1/6
**Sensineural hearing loss**	No	No	No	No	Yes	No	1/6
**Hepatoplenomegaly**	Yes	Yes	Yes	Yes	Yes	No	5/6
**Eczema**	Yes	No	Yes	No	No	Yes	3/6

Supplementary Table 1. Features of Roifman Syndrome for the affected individuals in this study.

	Patients					
	**1**	**2**	**3**	**4**	**5**	**6**
Lymphocyte Markers x10g/L						
**CD3**	1.6	2.3	2.2	3.1	2.4	2.66 (0.66-2.41)
**CD4**	1.0	1.4	1.7	1.4	1.6	1.63(0.43-1.62)
**CD8**	0.6	0.9	0.4	1.2	0.7	1.03(0.15-1.01)
**CD4/20**	0.07	0.08	0.04	0.04	0.39	0.07(0.08-0.58)
**CD16/56**	0.2	0.29	0.18	0.32	0.58	0.11(0.05-0.52)
Mitogen Response						
**PHA (counts$X10^{-3}$)**	123(95)	119(117)	105(130)	Normal	Normal	ND
**SAC**	3.3(18)	2.6(14)	4.1(21)	ND	ND	ND
**Immunoglobulins(g/L)**						
**IgG**	17.90(6.7-17)	10.00(6.7-17)	9.50(6.7-17)	13.5	2.33(>4.2)	4.3(7.0-14.0)
**IgA**	6.77(0.4-3.7)	1.93(0.4-3.7)	2.50(0.4-	1.21	0.31	3.8(0.4-1.9)

			3.78)			
**IgM**	0.20(0.5-3)	0.83(0.5-3)	1.10(0.5-3)	0.37	0.41(>0.43)	0.6(0.5-1.6)
**IgE**						10(?)
Specific antibodies						
**Anti-Tetanus (IU/ml)**	<0.01(>0.04)	<0.01(>0.04)	<0.01(>0.04)	0.27	0.51	<0.1(>0.16)
**Anti-Polio**	<1/8(>1/16)	<1/8(>1/16)	<1/8(>1/16)			
**Anti-Diptheria(IU/ml)**						0.03(>0.1)
**Anti-Haemophilus Influenzae(IU/ml)**				0.23	1.02	<0.11(>0.15)
**Anti-Pneumococcus (fold increase)**	NR(>3)	NR(>3)	NR(>3)	Borderline	very poor	

Supplementary Table 2. Immunological findings for the affected individuals in this study.

	**Aff. sibling 1**		**Aff. sibling 2**	
	**bp**	**bp percent.**	**bp**	**bp percent.**
**depth == 0**	41,834,894	1.47%	41,452,314	1.45%
**uniquely aligned reads, 5 > depth >= 1**	38,701,534	1.36%	36,867,390	1.29%
**uniquely aligned reads, 151 > depth >= 5**	2,767,536,543	97.17%	2,770,696,902	97.25%

Supplementary Table 3. Coverage statistics for Complete Genomics whole genome sequencing of affected sibling 1 and 2 from kindred 1.

	**Aff. sibling 1**	**Aff. sibling 2**
**No filters**	4,070,206	4,086,111
**Quality tier >= 1**	3,689,435	3,720,277
**Quality tier == 2**	3,495,872	3,526,066
**Quality tier == 2, het-ref**	2,085,627	2,101,620
**Quality tier == 2, hom-alt**	1,327,154	1,344,848
**Quality tier == 2, chrX hap**	73,756	69,971
**Quality tier == 2, rare 1%**	56,914	57,013
**Quality tier == 2, rare 1%, Coding**	483	496
**Quality tier == 2, rare 1%,**	107	104

ncRNA		

Supplementary Table 4. Small variant (SNV and indel) counts for the two affected siblings. Abbreviations: quality tier >= 1 represents high and borderline quality, quality tier == 2 represents high quality; rare 1% represents variants with alternate allele frequency not exceeding 1% in any of the databases used; het-ref: heterozygous alternate allele; hom-alt: homozygous alternate allele; chrX hap: haploid (i.e. single copy) alternate allele on a male X chromosome. Please refer to Supplementary Note 6 for detailed definitions.

	**Aff. sibling 1**	**Aff. sibling 2**	**Both**
chrX hap	3	1	0
Hom	0	0	0
Potential compound het	8	6	2
Dominant gene	13	15	8
Novel, dominant gene	3	4	0

Supplementary Table 5. Prioritized variants (quality tier = 2, rare 1%, damaging) grouped by zygosity and gene mode of inheritance; for potential compound hets, we considered only variants from sets of quality tier-2 variants (i.e. potential compound hets with one quality tier-1 and one quality tier-2 variant were not considered). Please refer to Supplementary Note 7 for detailed definitions.

	UP_MIn	UP_MIy	UP_pv	UP_OR	UP_OR_CI1	UP_OR_CI2
fdr10	64	10	5.72E-05	5.242301353	2.389909914	10.33363437
fdr20	142	18	1.13E-06	4.286424137	2.455448206	7.071173791
pv0.05	722	81	1.73E-22	4.056884	3.14305872	5.18228584
NotSigFc2	864	10	0.000664012	0.375740111	0.178740075	0.698409437

Supplementary Table 6. Enrichment statistics showing that DESeq [9] up-regulated genes are significantly enriched in minor intron genes. Abbreviations: fdr10, fdr20: differential expression Benjamini-Hochberg FDR <= 10%, 20%; pv0.05: differential expression nominal p-value <= 0.05; NotSigFc2: genes with log2 (fold-change) >= 2 but nominal p-value > 0.05 (i.e. not significant); UP_MIn: number of up-regulated genes without minor introns; UP_MIy: number of upregulated genes with at least one minor intron; UP_OR, UP_OR_CI1, UP_OR_CI2:

enrichment Fisher’s Exact Test odds-ratio point estimate and 95% confidence interval (CI1, CI2).

	DW_MIn	DW_MIy	DW_pv	DW_OR	DW_OR_CI1	DW_OR_CI2
fdr10	92	2	1	0.720776362	0.085837998	2.688078022
fdr20	176	7	0.502805409	1.323087429	0.522396297	2.797785336
pv0.05	685	31	0.03153772	1.523865236	1.0194037	2.20331957
NotSigFc2	1880	27	1.42E-05	0.456913183	0.298103266	0.672799191

Supplementary Table 7. Enrichment statistics showing that DESeq down-regulated genes are not significantly enriched in minor intron genes. Abbreviations: same as Supplementary Table 6, with “DW” (down-regulated) replacing “UP” (up-regulated).

	UP_MIn	UP_MIy	UP_pv	UP_OR	UP_OR_CI1	UP_OR_CI2
fdr05	475	48	1.56E-12	3.597261489	2.58917318	4.904011142
fdr10	755	77	1.05E-19	3.755236296	2.891744916	4.82206984
fdr25	1382	109	1.57E-19	2.978180903	2.387497736	3.687869862
NotSigFc2	822	14	0.034319692	0.567444306	0.307161718	0.964357018

Supplementary Table 8. Enrichment statistics showing that cufflinks/cuffdiff [10] up-regulated genes are significantly enriched in minor intron genes. Abbreviations: fdr05, fdr10, fdr25: differential expression FDR q-value <= 0.05, 0.10, 0.25; NotSigFc2: genes with log2 (fold-change) >= 2 but nominal p-value > 0.05 (i.e. not significant); UP_MIn: number of up-regulated genes without minor introns; UP_MIy: number of upregulated genes with at least one minor intron; UP_OR, UP_OR_CI1, UP_OR_CI2: enrichment Fisher’s Exact Test odds-ratio point estimate and 95% confidence interval (CI1, CI2).

	DW_MIn	DW_MIy	DW_pv	DW_OR	DW_OR_CI1	DW_OR_CI2
fdr05	247	7	1	0.958291758	0.379828121	2.015059426
fdr10	387	9	0.646994637	0.783922606	0.354215247	1.51262954
fdr25	692	22	0.732128828	1.077918825	0.666484835	1.659219848
NotSigFc2	1711	28	0.000579175	0.534874726	0.351558084	0.783194944

Supplementary Table 9. Enrichment statistics showing that cufflinks/cuffdiff down-regulated genes are not significantly enriched in minor intron genes. Abbreviations: same as Supplementary Table 8, with “DW” (down-regulated) replacing “UP” (up-regulated).

	Diff_MIn	Diff_MIy	Diff_pv	Diff_OR	Diff_OR_CI1	Diff_OR_CI2
fdr05	256	99	6.09E-69	14.88052877	11.52450971	19.11932201
fdr10	369	123	8.21E-80	13.27664201	10.56633022	16.59690141
fdr25	694	167	6.15E-91	10.19799804	8.392970446	12.34680591
pv0.05	927	188	4.79E-92	8.843494363	7.357076495	10.60190482

Supplementary Table 10. Enrichment statistics showing that cufflinks/cuffdiff differentially spliced genes are significantly enriched in minor intron genes. Abbreviations: fdr05, fdr10, fdr25: differential expression FDR q-value <= 0.10, 0.25; pv0.05: differential splicing nominal p-value <= 0.05; UP_MIn: number of differentially spliced genes genes without minor introns; UP_MIy: number of differentially spliced genes with at least one minor intron; UP_OR, UP_OR_CI1, UP_OR_CI2: enrichment Fisher's Exact Test odds-ratio point estimate and 95% confidence interval (CI1, CI2).

	Gene N#	Wilcoxon P-value	Median log2ratio
Minor intron genes with any assembled isoform	593	2.81E-05	-0.001472046
Genes with at least one minor intron retention isoform	244	0.0002439	-0.01257166
Genes with at least one minor intron retention isoform significantly upregulated in affected subjects	32	0.03739	-0.02586186

Supplementary Table 11. Enrichment in down-regulation of isoforms without minor intron retention for minor intron genes, based on cufflinks isoforms. The enrichment Wilcoxon p-value was calculated by comparing the corresponding gene category to all other genes without minor introns.

**Threshold**	**Gene set**	**UP_MIn**	**UP_MIy**	**UP_pv**	**UP_OR**	**MI_tot**	**Intron_tot**
fdr10	All	155	92	1.68E-154	168.1	776	194213
fdr20	All	221	118	2.55E-194	156.8	776	194213
pv0.05	All	2262	285	0	49.0	776	194213
NotSigFc2	All	9036	35	0.931993108	1.0	776	194213
fdr10	Up-reg	127	33	5.12E-28	22.6	89	5102
fdr20	Up-reg	167	38	1.01E-30	21.6	89	5102
pv0.05	Up-reg	1046	66	2.75E-26	10.9	89	5102
NotSigFc2	Up-reg	399	7	1	1.0	89	5102
fdr10	Down-reg	0	1	0.003312549	Inf	34	10264
fdr20	Down-reg	1	1	0.006614446	303.4	34	10264

pv0.05	Down-reg	14	3	2.19E-05	70.3	34	10264
NotSigFc2	Down-reg	72	0	1	0.0	34	10264
fdr10	Unchanged	8	13	3.34E-26	395.3	137	30494
fdr20	Unchanged	10	19	1.31E-38	492.9	137	30494
pv0.05	Unchanged	124	53	1.33E-84	153.5	137	30494
NotSigFc2	Unchanged	540	2	1	0.8	137	30494
fdr10	MI_Signif	25	92	2.21E-90	64.6	776	12826
fdr20	MI_Signif	36	118	6.80E-114	59.7	776	12826
pv0.05	MI_Signif	382	285	4.11E-183	17.7	776	12826
NotSigFc2	MI_Signif	452	35	0.285716782	1.2	776	12826
pv0.1_fc1.5	MI_EffectSize	675	337	1.22E-179	12.9	776	12826
pv0.1_fc2.0	MI_EffectSize	566	319	1.34E-180	14.2	776	12826
pv0.1_fc3.0	MI_EffectSize	202	220	2.12E-161	23.2	776	12826
pv0.1_fc4.0	MI_EffectSize	98	146	1.28E-116	28.2	776	12826

Supplementary Table 12. Enrichment in minor intron up-regulation for minor intron genes, based on intron DESeq analysis. Threshold: fdr10, fdr20: differential expression significance threshold BH-FDR <= 10%, 20%; pv0.05: differential expression significance threshold nominal p-value <= 0.05; NotSigFc2: introns with log2 (fold-change) >= 2 and p-value > 0.05; Gene set: gene subset (all, up-regulated, down-regulated, unchanged, MI genes stratified by significance, MI genes stratified by effect size); UP_MIn: number of up-regulated major introns; UP_MIy: number of up-regulated minor introns; UP_pv: FET nominal p-value for minor intron enrichment in intron up-regulation; UP_OR: FET odds ratio point-estimate for minor intron enrichment in intron up-regulation; MI_tot: total number of minor introns in the DESeq output; Intron_tot: total number of introns in DESeq output.

IsoformCat	sample1	sample2	WcxPvalue_Less	WcxPvalue_Great
IntronRetention_MIy	84	80	1	7.64E-14
IntronRetention_MIy	83	81	1	1.55E-25
IntronRetention_MIy	83	82	1	5.82E-25
known	84	80	1.84E-09	0.999999998
known	83	81	2.19E-17	1
known	83	82	9.04E-12	1

Supplementary Table 13. Enrichment statistics for iReckon [11] isoforms and minor introns, part 1: affected subjects display significantly higher RPKM for minor intron retention isoforms and significantly lower RPKM for known isoforms, compared to family-matched unaffected subjects. Abbreviations: IsoformCat: isoform category (“IntronRetention_MIy”, “known”), sample1, sample2: sample pair used for the comparison (sample1: affected, sample2: unaffected), WcxPvalue_Less: Wilcoxon paired test one-sided p-value, significant when “sample1 << sample2”, WcxPvalue_Greater: Wilcoxon paired test one-sided p-value, significant when “sample1 >> sample2”.

IsoformCat	Log2rRPKM_Q1	Log2rRPKM_Q2	Log2rRPKM_Q3
IntronRetention_MIy	0	0	17.37472229
IntronRetention_MIy	0.048705805	17.06171405	18.60186065
IntronRetention_MIy	1.258688678	17.186478	18.57205468
known	-1.041333473	-0.271538916	0
known	-1.528940546	-0.823278817	0
known	-0.971210176	-0.391927374	0

Supplementary Table 14. Enrichment statistics for iReckon isoforms and minor introns, part 2: corresponding RPKM quartiles (Q1, Q2, Q3). Abbreviations: IsoformCat: same as Supplementary Table 13, Log2rRPKM_Q1, Log2rRPKM_Q2, Log2rRPKM_Q3: 25% distribution quantile, median and 75% distribution quantile of the Log2 RPKM ratio (affected / unaffected).

	+/-100 bp padding added	RNU4ATAC Covered
LifeTech AmpliSeq 20131001	no	0%
Nextera RapidCapture Expanded v1.2	yes	100%
Nextera RapidCapture v1.2	yes	100%
Nimblegen SeqCapEZ v2	yes	0%
Nimblegen SeqCapEZ v3	yes	0%
Nimblegen SeqCapEZ UTR v3	yes	0%
SureSelect HumanAllExon V4	yes	0%
SureSelect HumanAllExon +UTRs V4	yes	0%
SureSelect HumanAllExon 50Mb	yes	0%
SureSelect HumanAllExon V2	yes	0%
SureSelect HumanAllExon V5	yes	0%
SureSelect HumanAllExon V5 and Clinical	yes	0%
SureSelect HumanAllExon +UTRs V5	yes	0%
Truseq exome	yes	100%

Supplementary Table 15. *RNU4ATAC* coverage for commercial exome capture kits.

Subject ID	Kindred, Patient index	Role	Cond.	Gender, Age	Trimmed read pairs	rRNA % read pairs	concord. aligned % read pairs
83	k1.p2	son	aff.	M, 38	31,708,037	8.59%	87.0%

82	k1	son	unaff.	M, 43	38,088,242	4.26%	91.5%
81	k1	father	unaff.	M, 67	27,971,870	12.80%	82.6%
84	k2.p3	son	aff.	M, 21	27,983,435	3.74%	91.2%
80	k2	father	unaff.	M, 57	32,597,742	9.60%	85.0%

Supplementary Table 16. Subject information, RNA-seq read number and alignment statistics.

Mut.	Sub#N	Zyg.	C.het.Link	Severity	Life exp.	Cause of D.	Pop.	Ref.
51 G>A	7	hom	NA	most severe	2.5-18 months	NA	Ohio Amish	He et al. Science 2011 [12]
51 G>A	1	hom	NA	most severe	33 days	failure to thrive	Australian / Maltese	He et al. Science 2011 [12], Haan et al 1989 AJMG [13]
51 G>A	2	hom	NA	most severe	10 months, 11 months	infectious / neurological	Algerian	Edery et al. Science 2011 (F1) [14]
51 G>A	1	hom	NA	most severe	14 moths	infectious	Turkish	Edery et al. Science 2011 (F2) [14]
51 G>A	1	hom	NA	most severe	7 months	infectious	Moroccan	Edery et al. Science 2011 (F3) [14], Sigaudy et al 1998 AJMG [15]
51 G>A	3	hom	NA	most severe	1 month, 28 months, abortion	infectious, abortion	Moroccan	Edery et al. Science 2011 (F4) [14]
51 G>A	1	hom	NA	most severe	NA	NA	Indian	Edery et al. Science 2011 (F5) [14]
50 G>A	1	comp.het	1	most severe	6 months	NA	North American Caucasian	Edery et al. Science 2011 (F6) [14]
51 G>A	1	comp.het	1	most severe	6 months	NA	North American Caucasian	Edery et al. Science 2011 (F6) [14]
50 G>C	1	comp.het	2	severe	28 months	NA	North American Caucasian	Edery et al. Science 2011 (F7) [14]
51 G>A	1	comp.het	2	severe	28 months	NA	North American Caucasian	Edery et al. Science 2011 (F7)

								[14]
51 G>A	1	comp.het	3	most severe	abortion	abortion	Norwegian	Edery et al. Science 2011 (F8) [14]
53 C>G	1	comp.het	3	most severe	abortion	abortion	Norwegian	Edery et al. Science 2011 (F8) [14]
55 G>A	1	hom	NA	mod. severe	survival past 9 years	NA	German	He et al. Science 2011 [14], Nagy et al. CG 2011 [16]
55 G>A	2	hom	NA	severe	18 months, 34 months	infectious	Yemeni	Abdel-Salam et al. AJMG 2012 [17]
55 G>A	2	hom	NA	mod. severe	survived past 5 years, past 2.5 years	NA	NA	Abdel-Salam et al. AJMG 2011 [18]
30 G>A	1	comp.het	4	least severe	12 years 3 months	infectious	German	He et al. Science 2011 [14], Klinge et al N 2002 [19], Nagy et al. CG 2011 [16]
111 G>A	1	comp.het	4	least severe	12 years 3 months	infectious	German	He et al. Science 2011 [14], Klinge et al N 2002 [19], Nagy et al. CG 2011 [16]
66 G>C	1	comp.het	5	least severe	NA	NA	Egyptian	Abdel-Salam et al. AJMG 2012 [17]
66 G>C	1	hom	NA	mod. severe	survived past 3 years	NA	Turkish	Kilic et al. AJMG 2015 [20]

Supplementary Table 17. MOPD1 published causal variants and degree of severity. Note that when the same mutation was found in more than one affected individual from the same kindred, information about life expectancy and cause of death is separated by comma (e.g. 10 months, 11 months), if more individuals were affected from an extended pedigree, information may be provided as a range.

SUPPLEMENTARY NOTES

SUPPLEMENTARY NOTE 1: PATIENT INFORMATION

Patient 1 (Kindred 1)

Patient 1 was born at 35 weeks gestation to non-consanguineous parents of English descent. His birth weight and length plotted below the 3rd centile for his gestational age. Birth head circumference measurement was not available.

The patient suffered repeated upper respiratory and ear infections, and required myrigotomy tube insertion at the age of 2 ½ years. He continued up to the age of 5 years to experience febrile episodes once per month and had multiple admissions to the hospital for bacterial pneumonias. At the age of 6 years, he was admitted for high fever, hepatosplenomegaly and progressive respiratory failure. Chest radiography showed bilateral lung consolidations. He was diagnosed with pneumonia and septicemia and improved after treatment with piperacillin and tobramycin. Throughout his childhood, the patient suffered from moderate atopic dermatitis and repeated episodes of Herpes Simplex Virus (HSV) infections with lesions at the right maxillary ridge and right frontal areas.

Upon immunological assessment for his history of recurrent infections, the boy was diagnosed with antibody deficiency (see Supplementary Table 2) and prescribed monthly IVIg therapy, which reduced the infection frequency.

At the age of 14 years, the patient was admitted to hospital for repeated episodes of vomiting, decreasing exercise tolerance and fatigue over a period of 3 months. Physical exam and related investigations revealed a gallop rhythm, hepatomegaly and pulmonary edema. Electrocardiogram showed left ventricular hypertrophy and echocardiogram revealed severe left ventricular dysfunction with ejection fraction of 17%; the heart wall movement showed a pattern typical of non-compaction. The boy's symptoms resolved gradually in response to treatment with furosemide and digoxin [21].

The patient had global developmental delay and mild generalized hypotonia. He sat at eight months and walked at 2 years. His speech was also delayed. At the age of 7 years, the patient underwent psycho-educational assessment. His intelligence could not be assessed by the Wechsler scales because of his inability to co-operate. Instead, the Leiter test was used and showed that the patient's intellectual ability was equivalent to that of a 4 year old (< 1st centile). Single word comprehension and expression as well as grammatical comprehension were at the 5 year old level. Basic concepts fundamental to understanding verbal instructions measured at the 3rd centile.

Head circumference plotted below the 3rd centile (-3SD) when he was assessed at the age of 17 years. MRI brain was performed and was normal.

That patient's weight and height continued to track below the 3rd centile for age and final adult height was 3-4 SD below the mean. Adult weight plotted 2-3 SD below the mean. Skeletal survey was performed and showed spondyloepiphyseal dysplasia and shortened metacarpals. Eye exam was performed in light of the diagnosis of Roifman syndrome, and was normal.

Patient 2 (Kindred 1)

Patient 2 is patient 1's older brother and was diagnosed following patient 1's evaluation. The boys also have an eldest brother who is healthy. Patient 2 was born at term birth weight and height below the 3rd centile. Birth head circumference was not known. He spent his first 11 days in the NICU because of slight breathing and feeding difficulties and jaundice.
During infancy and childhood, he suffered repeated episodes of pneumonia and ear infections. Myringotomy tubes were inserted at the age of 2 years and again at 12 years. Immunological work up revealed low number of circulating CD19+ B cells, but normal number and distribution of T cells. While serum IgG, IgA and IgM levels were normal, specific antibodies were universally markedly reduced.

An eye exam was performed at the age of 14 years because of vision concerns and revealed signs of retinal dystrophy.
Psychoeducational assessment performed at the age of 11 years because of academic difficulties showed he was functioning at a grade 1 level (4 grade levels below expected for his age) in math computations, reading comprehension, reading and spelling. At the age of 16 years a full scale IQ assessment was performed. The patient's major difficulties were with tasks requiring him to analyze abstract designs and construct them from blocks, solve puzzles and coding. Reading ability on the Woodcock test placed him at grade 3-4. Math assessment using the KeyMay Diagnostic Test showed competency levels at grade 2 or 3.
His weight was in the normal range in adulthood (10-25th centile) but his short stature remained (-3-4 SD). Head circumference in adulthood was within the normal range.
Skeletal survey showed spondyloepiphyseal dysplasia with shortened metacarpals.

Patient 3 (Kindred 2)
Patient 3 was born at 36 weeks via emergent Caesarian section due to placental abruption. His birth weight and height plotted below the 3rd centile. Birth head circumference measurement was not available.
The patient suffered repeated upper respiratory infection. Evaluation of his immune function at the age of 2 years, revealed low serum Ig and an inability to mount an antibody response after vaccination. He was given IVIg treatment which led to a slow decline in infection frequency.
The patient had one episode of autoimmune hemolytic anemia at age 2 ½ years following a flu-like illness. Direct Coombs test was positive and blood smear showed polychromasia with spherocytes and Howell-Jolly bodies. He was treated with a course of Prednisone and the anemia resolved 6 months later.
At the age of 7 years, the patient was admitted because of a 5-month history of chronic diarrhea. Gut biopsies obtained during endoscopy revealed signs of chronic colitis at multiple sites. The cecum and transverse colon showed reactive glandular changes; some of the glandular epithelium was infiltrated by neutrophils, eosinophils and lymphocytes; some of the glandular crypt bases had increased apoptosis; the descending colon showed reactive lymphoid follicle within the lamina propria; one mucosal gland contained an intra-epithelial infiltrated of eosinophils; and the rectum had signs of cryptitis and multiple mucosal gland infiltrates of neutrophils in the lamina propria. Immunostains for CMV and adenovirus were negative.
The patient had early gross motor delays, sitting at 11 months and walking at 22 months, with cognitive difficulties being more prominent later in his childhood. Psychological assessment was

performed when he was in Grade 4, and revealed both a learning disorder and Attention Deficit Hyperactivity Disorder – predominantly hyperactive/impulsive type. Cognitive functioning was tested using the Wechsler Intelligence Scale for Children (WISC-IV). His performance on the main areas of WISC-IV comprehension was at the 50th centile, perceptual reasoning at 5th centile, working memory at 21st centile, and processing speed at the 4th centile. He was found to be 'exceptionally weak' (identified as performance below the 3rd centile for age) in the following areas: skills in analyzing and organizing visual perceptual material; memory of meaningful visual information in recognizing changes or additions to scenes; and computational and math reasoning skills, (as measured by two subtests of the WIAT-II),

Head circumference measured at 17 years of age plotted just below the 3rd centile. MRI brain was normal.

His weight in adulthood was in the low-normal range (3-10th centile) and his height remained below the 3rd centile (-2-3 SD). Skeletal survey was performed because of short stature and brachydactyly; it showed signs of epiphyseal dysplasia, affecting the vertebrae, hips and metacarpals bilaterally.

Based on the features of humoral immune deficiency, cognitive delay and spondyloepiphyseal dysplasia, the diagnosis of Roifman syndrome was made. An eye examination was then performed and revealed signs of retinal dystrophy.

Patient 4 (Kindred 3)

Patient 4, a female, was born at 36 weeks gestation to non-consanguineous parents of Lebanese-Austrailian origin. Her weight, height and head circumference plotted at the 10th centile. Early on, she was noted to be mildly dysmorphic with brachydactyly of the hands and feet and hyper-extensible ligaments. A ventricular septal defect was also found.

During the neonatal period, she had a transient conjugated hyperbilirubinemia. While the jaundice settled, hepatosplenomegaly and elevated liver enzymes persisted. She therefore underwent liver biopsy, which was performed at the age of 3 ½ months. This showed expansion of the portal areas and sinusoids with extramedullary haematoposiesis including eosinophils, neutrophils as well as B and T cells. In childhood, the patient suffered repeated discharging ear infections and recurrent oral thrush.

Her growth remained stunted, tracking below the 1st percentile for height and weight. Skeletal survey revealed epiphyseal dysplasia and shortened metacarpals. The vertebrae appeared normal.

She was also found to have mild conductive hearing loss. Eye examination was normal. She had generalized hypotonia.

The patient's cognitive delays were mild and did not come to attention until school age. Intellectual function, assessed by the WISC-IV test, showed mild intellectual impairment in verbal intelligence (0.5th percentile) and nonverbal intelligence (2nd percentile), borderline range processing speed (5th percentile) and low average working memory (9th percentile). Her ability to learn and remember structured verbal material was borderline and her memory for unstructured verbal material was average.

Patient 5 (Kindred 3)

Patient 5, the younger brother of patient 4 was born at term with weight at the 2nd centile, length below the 3rd centile (-3-4 SD) and head circumference below 3rd percentile. He, too, suffered neonatal jaundice and hepatosplenomegaly, and when the jaundice subsided he continued to have a larger liver and spleen. Evaluation by ultrasound demonstrated increased pressure within the portal tract, which was felt to indicate likely hepatic fibrosis. Phenotypically, he is similar to his sister with similar dysmorphic features (see Supplementary Table 1) and short stature (< 1st percentile). Distinct from his sister, he had retinal dystrophy, myopic astigmatism and marked sensorineural hearing loss considered sufficient to require hearing aids. His hypotonia and gross motor and speech delays were more severe than his sister's as well.
He suffered repeated infections, especially chest infections, since the age of 1 year. He had repeated pneumonias since the age of 2 ½ years, which were responsive to antibiotics.
Patient 5 had delayed motor and speech milestones. His psychological assessment (WISC-IV test) was performed at 7 years 4 months of age and showed mild intellectual impairment in verbal intelligence (0.5th percentile) and nonverbal intelligence (0.5th percentile) as well as processing speed (1st percentile) and borderline range working memory (3rd percentile). His overall level of intellectual functioning was in the range of mild intellectual impairment. Formal assessment of attention using the Test of Everyday Attention for Children showed extremely low ability to focus his attention on mundane tasks for an extended period of time. His ability to learn and remember structured verbal material was also poor.
Skeletal survey showed epiphyseal dysplasia with shortened metacarpals. The vertebrae were normal.

Patient 6 (Kindred 4)

Patient 6 is the youngest known case of Roifman syndrome at 4 years old. He was born at 37 weeks gestation to non-consanguineous parents of Albanian descent. His weight, length and head circumference measured <3rd percentile. He suffered mild neonatal jaundice.
From the age of 8 months he suffered repeated episodes of croup and at 9 months he had bronchiolitis. At 26 months of age he suffered pneumonitis. At 26 months of age he suffered pneumonia and had convulsions with fever. At 3 years he had a second episode of febrile convulsions during an episode of pneumonitis due to parainfluenza 3 and adenovirus. At 51 months, he had mycoplasma pneumonia and at 52 months metapneumovirus pneumonitis. At 53 months, he suffered perforation of his left tympanic membrane and purulent otorrhoea. He also had severe eczema, which was first noticed at 8 weeks of age over his trunk, arms and legs, which gradually improved with age. Evaluation of the immune system revealed low serum IgG with normal IgA and IgM, and low specific antibody levels to tetanus Haemophilus Influenzae and Diptheria Toxin. Lymphocyte markers revealed normal number of circulating T cells and NK cells but low numbers of CD19+ B cells.
Linear growth remained delayed at <3rd percentile, while weight was appropriate for age. Head circumference also remained below the 2nd percentile. Skeletal survey performed at 28 months demonstrated marked delay in ossification of the capital femoral epiphyses and flattening of acetabular roof. Both capital femoral epiphyses remained entirely cartilaginous. Knee epiphyses appeared fragmented and flattened bilaterally. Metatarsals and metacarpals appeared broadened. The metaphyses were normal and no abnormality was detected in the spine.

The boy’s development was mildly delayed with low to average performance. Formal cognitive assessments have not yet been performed. Head MRI and EEG were normal and eye examination showed normal retinas.

SUPPLEMENTARY NOTE 2: KINDRED 1 WHOLE GENOME SEQUENCING RESULTS

1. Coverage

For both subjects, > 97% of the genome was covered at depth >= 5 by uniquely aligned reads.

2. SNV and indel variant counts

The two affected siblings had a very similar number of total variants (no quality filter: 4,070,206 and 4,086,111, quality tier 2: 3,495,872 and 3,526,066) as well as gene-mapped, potentially damaging coding (quality tier 2: 483, 496) and non-coding variants (quality tier 2: 107, 104).

3. SNV and indel prioritization results, primary pipeline

None of the two siblings had any quality tier-2, 1% rare, potentially damaging homozygous variant.
The two siblings had several quality tier-2, 1% rare, potentially damaging X-linked variants, but none was shared. Since the X-linked mode of inheritance had been proposed, we investigated these particularly carefully. None of the coding variants present in only one of the two siblings were sufficiently damaging and disrupting a gene producing the expected phenotypic outcome: one of the two siblings had a missense damaging tier-1 variant on *SH3KBP1*, frequency 1-0.3%, and a missense damaging tier-1 variant on *AFF2*, frequency < 0.2%; *SH3KBP1* is implicated only in immune and metabolic abnormalities in mouse, while *AFF2* is implicated in intellectual disability in humans, but neither matches the full Roifman Syndrome phenotypic spectrum (growth, neurodevelopmental, bone and immune abnormalities). The other sibling had a very rare (< 0.6%) and damaging tier-2 X-linked variant on *ARSH* (arylsulfatase family, member H), which is not implicated in any human phenotype and does not have a mouse homolog. In addition, one of the siblings had a novel, X-linked non-coding variant disrupting the core splicing site of a poorly characterized antisense transcript gene (*HS6ST2-AS1*).
The two siblings had several quality tier 2, 1% rare, potentially damaging variants forming potential compound heterozygous sets (i.e. compound hets), but only one set of two quality tier-2 variants was shared, corresponding to the non-coding *RNU4ATAC* variants. Since several of the potential compound hets can be on the same phase (thus not forming authentic compound hets), it is not surprising that the prioritized variants found in both siblings are markedly fewer. Thanks to the short genomic interval between them, the phase of the *RNU4ATAC* variants was resolved by the variant calling algorithm, showing that the alternate alleles are on opposite phases. Notably, the variant mapping to the MOPD1-implicated 5’ stem loop is novel with respect to all frequency databases and dbSNP, while the 3’ stem loop variant is reported only for cgW597 (1000 Genomes subset on Complete Genomics) at frequency 0.0008, corresponding to only one allele in the 597 subjects set.

The two siblings also had 8 shared quality tier-2, 1% rare, potentially damaging variants on dominant genes, none of which was novel; three of these variants had frequencies below 0.1% (for the genes *HTT*, *RP1L1*, *GUCY2D*), and thus worth some more detailed investigation; however, only one of them was damaging tier-2 (for the gene *HTT*, whose known dominant mode of action is through toxic gain of function rather than loss of function), and none of them matched the expected phenotype. As far as the novel variants, which could have a de-novo origin, none was shared by the two siblings. Finally, there was no prior evidence that Roifman Syndrome could have a dominant mode of inheritance.
Notably, all prioritized variants shared between the two siblings were quality tier-2, further validating our thresholds for high quality.
For detailed results, please see Supplementary Data 1.

4. SNV and indel prioritization results, secondary pipeline

The two siblings shared 3 autosomal dominant classified as likely pathogenic variants, but none in the other likely pathogenic categories; in fact, all the other variants output by the secondary pipeline were heterozygous were classified as carrier status or mapped to genes with complex modes of inheritance; in addition, one shared variant was classified as forming a likely pathogenic potential compound het in one of the two siblings, but the other variant was not shared. The three heterozygous variants on dominant genes (*CYP2C19*, *SLC6A2*, *MC1R*) were at relatively high frequency (1% or higher) and did not match the expected phenotype. The *RNU4ATAC* compound het was not found by this pipeline, as it only considers loss-of-function (stop-gain, frameshift, splicing) or known variants classified as pathogenic by HGMD or ClinVar. For detailed results, please see Supplementary Data 1.

5. Structural variants

Structural rearrangements reported in “highConfidenceSvEventsBeta” had 122 and 127 complex events, 1138 and 1177 deletions, 46 and 49 distal duplications, 2 and 1 interchromosomal events, 6 and 9 inversions, 19 and 25 probable inversions, 74 and 71 tandem duplications, for the two siblings respectively. Events were filtered to retain only the ones disrupting genes and supported by junctions never observed in the 54 unrelated control samples from the Complete Genomics diversity panel (pipeline version 2.0). This resulted in 21 and 20 events for the two affected siblings, with 9 genes disrupted by the same or similar events in both siblings (note that, unlike for SNV and indels, it is preferable to relax the exact overlap criterion to partial overlap). Most of the deletions were intronic, and none of the events severely disrupted the sequence of a gene that could account for the Roifman Syndrome phenotype (see Supplementary Data 3).

6. Copy number variants

The two affected siblings had 145 and 157 losses, and 135 and 139 gains respectively, with min CNV size 1,306 bp, median size 12,000 bp and max size 350,000 bp. Of these, 26 and 34 losses, and 32 and 32 gains, were not found in the 54 controls and had at least one gene with

exonic mapping in the two affected siblings, resulting in 36 genes with a rare exonic loss in both siblings and 27 genes with a rare exonic gain in both siblings. However, all rare exonic CNVs were found in the DGV (Database of Genomic Variants) [22] and/or were completely within a segdup or overlapped other repeat-rich sequence; none of them could account for the Roifman Syndrome phenotype based on the overlapped genes (see Supplementary Data 2).

SUPPLEMENTARY NOTE 3: RNU4ATAC VARIANT IN OTHER GENOME COHORTS

1. RNU4ATAC variants in internal whole genome controls

We analyzed the following whole genomes, sequenced for diverse research projects unrelated to Roifman Syndrome or MOPD1:

- 528 Complete Genomics genomes (assembly version 2.0-2.4) received by The Centre for Applied Genomics (TCAG) up to May 2014, including single unrelated individuals, autism quads and a few extended pedigrees; 31 genomes carried only one *RNU4ATAC* heterozygous variant each, with none carrying homozygous or potential compound heterozygous variants.
- 98 autism trio whole genomes (for a total of 294 subjects), sequenced on Illumina HiSeq2000 by BGI (Beijing Genomics Institute), at depth 35-40x; 2 fathers, 4 mothers, 3 probands (for a total of 6 trios) had at least one *RNU4ATAC* variant; all subjects but one carried only one heterozygous variant each; one subject (a mother of an autism proband, not reported to have any clinical abnormality) carried two *RNU4ATAC* heterozygous variants (chr2:122288468:C>T, chr2:122288549:A>G); since neither variant was inherited by her autistic child, the two variants were inferred to be on the same phase, thus not constituting a compound heterozygous set; even in case of true compound heterozygosity, one of the two variants (chr2:122288549:A>G) is at a poorly conserved position within the dispensable region of the 3' stem-loop (94), predicted to have no functional effect on minor intron splicing.

2. Estimated prevalence of RNU4ATAC genetic disorder

We extracted all *RNU4ATAC* single nucleotide variants from the three allele frequency control databases based on whole genome sequencing (1000 Genomes, Complete Genomics Wellderly and Complete Genomics 1000 Genomes subset). We transformed allele frequencies to subject counts by multiplying the allele frequencies to the allele total (1092 * 2, 597 * 2, 436 * 2, respectively). For each position corresponding to (a) disease variants (Roifman Syndrome and MOPD1), or (b) disease variants and conserved positions within elements critical for splicing (see main text figure 3), we then added up the corresponding variant subject count from each database, and divided by the subject grand total, to finally obtain the frequency of heterozygous variant carriers in the general population, i.e. the probability of a subject from the general population being a heterozygous variant carrier. We can assume none of the subjects is homozygous or compound heterozygous, as he/she would otherwise develop disease (in addition, for Complete Genomics datasets genotype counts are available, and none is

homozygous). These estimates were also calculated excluding the 1000 Genomes dataset, which has lower whole genome sequencing depth.
The probability of a pregnancy presenting a bi-allelic *RNU4ATAC* alteration was estimated as ¼ of the square of the sum of all variant heterozygous carrier probabilities; assuming each variant belongs to a separate haplotype, probabilities at single positions represent separate events; since frequencies are typically < 1%, the probability of combined events (homozygosity, multiple heterozygous positions) is negligible, and thus (in first approximation) we can simply sum the probabilities of heterozygosity without removing the probabilities of combined events. This resulted in the probability 7.80E-06 when considering only disease variants (1.82E-05 when excluding 1000 Genomes), and 1.57E-05 when also considering conserved positions within splicing-critical elements (3.25E-05 when excluding 1000 Genomes), leading to a final estimate of 0.78-3.25 / 100,000 pregnancies (i.e. up to 1 in 30,000).

SUPPLEMENTARY NOTE 4: MOPD1 AND OTHER MINOR SPLICEOSOME DISORDERS

MOPD1 is characterized by severe intrauterine and postnatal growth retardation; short, bowed long bones with severe delay in epiphyseal maturation; severe microcephaly; brain malformation including pachygyria and corpus callosum agenesis; cardiac abnormalities; dysmorphic facial features and sparse hair; stillborn or premature death within the first year(s) of life [15, 23-25]. Most often, patients die because of infectious diseases [15], although severe neurological and endocrine abnormalities have also been reported as the cause of death [25]. MOPD2 and MOPD3 are syndromes with related phenotypic presentation. MOPD2 is caused by recessive causal variants of *PCNT* (pericentrin, entrez-gene id 5116, required for centrosome function), and does not present immunodeficiency. The syndrome originally designated MOPD3 was later recognized to be a phenotypic variation of MOPD1, and the few reported cases presented death in the first years of life due to infections [26-27] or even more premature death due to failure to thrive [13]. In contrast, and probably adding to the confusion, when Majewski later recognized that the originally defined MOPD3 cases should be classified as MOPD1, he also proposed the use of “MOPD3” for other cases of primordial dwarfism with severe intellectual disability, but no immunodeficiency or death in the first years of life, including the case of Caroline Crachami (also known as the Sicilian fairy) [28]. We think this type of MOPD3 is less likely to be caused by RNA4ATAC causal variants, or could be caused by RNA4ATAC causal variants with reduced penetrance, in a different domain than MOPD1 causal variants.

Besides MOPD1 and Roifman Syndrome, specific deficit of the minor spliceosome function has been reported only for isolated familial growth hormone deficiency, caused by compound heterozygous causal variants in the *RNPC3* protein-coding gene [29]. This suggests growth retardation, intellectual disability and bone defects as common outcomes of minor spliceosome dysfunction, though with different degrees of severity and specific presentations. Additional disorders are caused by defective minor intron splicing of specific genes and result in organ or even cell type-specific alterations (e.g. spondyloepiphyseal dysplasia tarda is caused by *SEDL* minor intron splice site causal variants, entrez-gene id 6399) [1].

Strikingly, of all the minor spliceosomal snRNAs, only *RNU4ATAC* has been implicated in Mendelian disorders. Since minor snRNAs (U4atac, U6atac, U11, U12) are expressed from single loci in the genome, homozygous or compound heterozygous loss-of-function causal variants in these snRNA genes could disable the minor spliceosome function as demonstrated for *RNU4ATAC* [1]; however, disease-implicated variants may have been missed by exome studies that did not include snRNAs in the captured genomic regions. Furthermore, seven proteins have been reported as specific to the minor spliceosome; even though their knockdown has been shown to interfere with human cell viability [30-31], only *RNPC3* recessive causal variants have been implicated in Mendelian disorder. This suggests that more Mendelian disorders caused by spliceosomal defects may be discovered, further extending the associated phenotypic spectrum. Disruption of protein components may result in less severe outcomes, or less widespread organ abnormalities, compared to snRNAs disruption.

SUPPLEMENTARY NOTE 5: RNA-SEQ ANALYSIS RESULTS

1. Alignment, QC, and summary of results (cufflinks, DESeq, iReckon)

The number of trimmed reads was comprised in the 27,971,870 - 38,088,242 range. The human rRNA content was comprised in the 3.74% - 12.8% range (of the trimmed reads). Concordantly aligned reads were in the 82.6 - 91.5% range (of the trimmed reads) (see Supplementary Table 16).

Cluster analysis confirmed separation of affected and unaffected samples (see Supplementary Fig. 4).

Cufflinks [10] assembled novel transcripts with minor intron retention for 244 genes; intron retention transcripts were expressed at significantly higher levels in affected subjects for 32 genes, while they were significantly higher in unaffected subjects for only one gene (differential isoform nominal p-value <= 0.05). For these 244 genes, the most expressed physiological transcripts were expressed at reduced levels in affected subjects, a significant trend compared to genes without minor introns (Wilcoxon test p-value < 1E-04, see supplementary results section D4 and D5).
iReckon [11] detected a similar number of minor intron retention transcripts, with significantly higher expression levels in affected subjects (Wilcoxon paired test p-value < 1E-13), and a corresponding decrease in physiological transcripts (Wilcoxon paired test p-value < 1E-08, see supplementary results section D7).
Specific retention of minor rather than major introns was confirmed in affected subjects using DESeq [9] (Fisher's Exact Test p-value < 1E-100, see supplementary results section D6).
In contrast, gene-level expression analysis (which does not distinguish different transcript categories) showed that minor intron genes have increased levels in affected subjects, with a significant enrichment compared to other genes (Fisher's Exact Test p-value < 0.0001, see supplementary results section D2 and D3).

2. DESeq differential gene expression analysis

Genes carrying a minor intron were significantly enriched in up-regulated genes at different significance thresholds for differential expression (Benjamini-Hochberg FDR adjusted p-value <= 0.10, <= 0.20 and nominal p-value <= 0.05) (see Supplementary Table 6), but they were not significant or just borderline significant for enrichment in down-regulation (same significance thresholds for differential expression, see Supplementary Table 7); up-regulation here is used to indicate higher levels in affected compared to unaffected, and vice-versa for down-regulation. Interestingly, up-regulation enrichment p-values increased in significance when relaxing the differential expression significance threshold, although the odds ratio moderately decreased. As a negative control, we tested enrichment in up-regulated genes with log2 (fold-change) >= 2 but nominal p-value > 0.05, finding a significant depletion. Altogether, these results suggested that noisy differential expression is not driving the up-regulation enrichment.
Enrichment was tested using the two-sided Fisher's Exact Test (FET), with contingency table counts defines as: (a) significantly up-regulated (or down-regulated) minor intron genes, (b) significantly up-regulated (or down-regulated) genes without minor introns, (c) minor intron genes with no significant change in the DESeq output, (d) genes without minor introns with no significant change in the DESeq output.

Total number of minor intron genes for DESeq gene differential analysis: 742
Total number of genes for DESeq gene differential analysis: 25,369

3. Cufflinks/cuffdiff differential gene expression analysis

Cufflinks/cuffdiff produced more significant differentially expressed genes than DESeq at corresponding significance thresholds. Nonetheless, we found a similar enrichment of minor intron genes in significantly up-regulated but not in significantly down-regulated genes; similar to DESeq, minor intron genes were also depleted of up-regulated genes with log2 (fold-change) >= 2 but nominal p-value > 0.05 (see Supplementary Tables 8-9).
For differential expression significance thresholds producing a similar number of DESeq and cufflinks/cuffdiff up-regulated genes (DESeq: nominal p-value <= 0.05, cufflinks/cuffdiff: FDR q-value <= 0.10), we found slightly higher enrichment odds ratios for DESeq (DESeq: odds ratio = 4.06, cufflinks/cuffdiff: odds ratio = 3.76), suggesting that DESeq differential results are more stringent than cufflinks/cuffdiff (see Supplementary Tables 6-9).
Adjusting for different stringency, we found a reasonable overlap between DESeq and cufflinks/cuffdiff up-regulated genes: for the genes without minor introns, 320 of the 722 DESeq and 755 cufflinks/cuffdiff overlapped; the overlap was greater for the up-regulated genes with minor introns: 50 of the 81 DESeq and 77 cufflinks/cuffdiff.

Total number of minor intron genes for cufflinks gene differential analysis: 695
Total number of genes for cufflinks gene differential analysis: 24,206

4. Cufflinks/cuffdiff differential splicing output analysis

Genes carrying a minor intron were significantly enriched in differentially spliced genes at different significance thresholds for differential splicing output (FDR q-value <= 0.05, <= 0.10,

<= 0.25 and nominal p-value <= 0.05). Enrichment p-values increased in significance when relaxing the differential splicing significance threshold, while the odds ratio decreased as expected (see Supplementary Table 10).

Total number of minor intron genes for cufflinks gene differential analysis: 727
Total number of genes for cufflinks gene differential analysis: 25,162

5. Cufflinks isoform intron retention analysis

Cufflinks assembled novel transcript isoforms (transcript class code "j") with minor intron retention (at least 25% of a minor intron overlapped) for 244 genes; intron retention isoforms were expressed at significantly higher levels in affected subjects for 32 genes, while they were significantly higher in unaffected subjects for only one gene (cufflinks differential isoform nominal p-value <= 0.05).

For these 244 genes, the most expressed physiological transcript isoforms were expressed at lower levels in affected subjects, a significant trend if compared to genes without minor introns. For this analysis, we categorized transcript isoforms in three groups: novel with minor intron retention (cufflinks isoform code "j" and at least 25% of a minor intron overlapped), known without minor intron retention (cufflinks isoform code "=" and no overlap with minor or major introns), other (isoforms not matching any of the other two definitions, thus having major intron retention, limited minor intron retention, or not matching known isoforms for other reasons). For each gene, the most expressed physiological transcript isoform was identified as the known isoform with the highest FPKM in the unaffected subjects, relative to other known isoforms. Enrichment in down-regulation of the most expressed physiological transcript isoform in the 244 genes with minor intron retention compared to other genes (excluding any gene with minor introns) was tested using the Wilcoxon one-sided test to compare the log2 fold-change; this resulted in a p-value of 1.402e-05.

We additionally investigated down-regulation of isoforms without intron retention, for genes with minor intron retention. We classified all isoforms in three groups: (i) minor intron retention (overlapping at least 25% of a minor intron), (ii) dubious (overlapping < 25% of a minor intron), (iii) without minor intron retention, regardless of other intron retention events and cufflinks isoform class code. Then, for every gene, we generated FPKM totals for isoform group (i) and (iii) by summing the average FPKM values for the affected and unaffected subject; to stabilize the FPKM ratio between affected and unaffected samples, zero values were replaced with 0.001 (roughly corresponding to the minimum non-zero value) and genes with FPKM for both groups and conditions below the median FPKM value were discarded. Minor intron genes with any assembled isoform (593 genes), genes with at least one minor intron retention isoform (244 genes) and genes with at least one minor intron retention isoform significantly upregulated in affected subjects (32 genes) all presented a small in absolute terms albeit significant negative shift of the log2 (affected / unaffected) FPKM ratio relative to the isoform group without minor intron retention, compared to other genes (see Supplementary Table 11). The shift significance was tested using the two-sided Wilcoxon's test.

6. DESeq differential intron expression analysis

Of 203,456 processed introns in the DESeq differential results, 9,235 corresponded to genomic loci with overlapping multiple genes and were ignored for further analysis. In the resulting DESeq differential output table, 776 unique introns had a match to a minor intron.
Compared to major introns, minor introns were extremely enriched in the up-regulated introns, when considering all genes (p-value < 1E-100, odds ratio > 40), and also when considering only up- or down-regulated genes. An even more extreme enrichment effect size (odds ratio > 150) was found when considering only unchanged genes (DESeq gene differential expression nominal p-value >= 0.1, absolute log2 (fold change) < log2 (1.1), corresponding to 2,975 genes); this was probably the case because unspecific intron up-regulation tied to overall gene up-regulation is not present for such genes. Finally, when only minor intron genes were considered, we still found a strong enrichment in minor intron up-regulation compared to major introns. We additionally demonstrated that the enrichment was present when defining up-regulated introns based on DESeq significance (p-value, BH-FDR) as well as when defining up-regulated introns based on DESeq fold-change (i.e. the effect size as opposed to the significance of intron up-regulation) (see Supplementary Table 12).

7. iReckon intron retention analysis

iReckon isoforms were classified based on 7 mutually exclusive categories: (a) known isoform with intron retention involving at least one minor intron (“IntronRetention_MIy”), (b) known isoform with intron retention involving only major intron(s) (“IntronRetention_MIn”), (c) canonical known isoform (“known”), (d) novel isoform without intron retention (“novel”), (e) novel isoform with intron retention involving at least one minor intron (“NovelIntronRetention_MIy”), (g) novel isoform with intron retention involving only major intron(s) (“NovelIntronRetention_MIn”), (h) unspliced pre-mRNA (“pre-mRNA”). Categories were constructed by parsing iReckon isoform names, matching the tags “IntronRetention”, “novel”, “unspliced”; in addition, isoform exons were matched to minor intron coordinates, to further categorize intron retention events as involving at least one minor intron or only major introns.
Then, for each gene and each subject, isoform RPKM values were summed to obtain category totals, resulting in 7 RPKM values per gene. RPKM tables for different subjects were merged, replacing missing values with RPKM = 0. The merged table had 22,580 genes, including 538 / 744 minor intron genes. More genes had “IntronRetention_MIy” isoforms in affected subjects compared to unaffected (affected: 158, 96; unaffected: 13, 16, 16). The RPKM for that category were accordingly shifted to higher levels for affected compared to unaffected
To more systematically explore the relative representation of different isoform categories we transformed absolute RPKM to percentage RPKM: for each subject and for each gene, RPKM for each category were divided by the total gene RPKM. We found a major difference between affected and unaffected “IntronRetention_MIy”, which was followed up by more detailed tests, while other differences were less pronounced and did not correlate with the affected / unaffected condition and thus might reflect an isoform detectability bias (see Supplementary Figure 5).

Since iReckon does not provide a native differential expression analysis for genes and isoforms (unlike cufflinks), we decided to compare aggregate abundance estimates (RPKM) for different isoform groups, for each affected-unaffected pair. Therefore, for each affected-unaffected subject pair, we tested the subset of genes with detected “NovelIntronRetention_MIy” isoforms in any of the affected or unaffected subjects, demonstrating that (i) the “NovelIntronRetention_MIy” category RPKM is significantly higher in affected subjects, (ii) the “known” category RPKM is significantly lower in affected subjects. We used two one-sided paired Wilcoxon t-test, where genes were used to pair RPKM values from each affected and unaffected subject tested (see Supplementary Table 13-14).

SUPPLEMENTARY NOTE 6: SNV AND INDEL ANNOTATION

1. Annotation overview

Complete Genomics masterVar files were annotated using a custom pipeline based on Annovar, producing annotated variant tables including these information categories:

- coordinates
- allele sequence and ploidy
- quality scores and read counts
- allele frequencies and variation databases
- gene mapping and coding sequence effect
- impact prediction and conservation

Complete Genomics file format documentation, including details about the masterVar file format, can be found here:
http://www.completegenomics.com/documents/DataFileFormats_Standard_Pipeline_2.0.pdf

2. Coordinates

chr: chromosome (autosomes 1-22 and sex chromosomes X, Y);
start: start position (1-positional system);
end: end position (1-positional system);
locusid: Complete Genomics unique variant identifier.

3. Allele sequence and ploidy

zygosity**:** heterozygous-reference (het-ref), homozygous-alternate (hom); heterozygous-alternate (het-alt), haploid-alternate (hap); the latter are on chromosomes that exist only in one copy (e.g. male sex chromosomes, X, Y);
reference: reference allele sequence;
allele1: allele 1 sequence; this typically corresponds to the alternate allele;
allele2: allele 2 sequence; this typically corresponds to the reference allele for het-ref variants;
Genotype: the genotype, represented as “reference allele | alternate allele” (het-ref) or “alternate allele | alternate allele” (hom), or “alternate allele” (hap), or “alternate allele 1 | alternate allele 2”

(het-alt);
allele1Haplink: numerical code identifying the phase of allele 1; two alleles are in phase when they have the same haplink code;
allele2Haplink: numerical code identifying the phase of allele 2; two alleles are in phase when they have the same haplink code;
varType: snp (single nucleotide variation, can be het-alt), ins (insertion), del (deletion), sub (block substitution, can differ in length from ref, but cannot be het-alt), complex (het-alt block substitution);

4. Quality scores and read counts

allele1ReadCount, allele2ReadCount: number of reads supporting allele 1 and allele 2, respectively;
referenceAlleleReadCount: number of reads supporting the reference allele (this may be equal to allele2ReadCount if allele 2 represents the reference);
totalReadCount**:** total read count;
allele1VarScoreVAF, allele2VarScoreVAF: quantitative quality score in Phred scale for allele 1 and allele 2, respectively, based on the VAF model (Variant Allele Fraction), which does not penalize allele fractions deviating from the diploid model;
allele1VarScoreEAF, allele2VarScoreEAF: quantitative quality score in Phred scale for allele1 and allele 2, respectively, based on the EAF model (Equal Allele Fraction), which penalizes allele fractions deviating from a diploid model;
Allele1VarQuality, Allele2VarQuality: default quality filter for allele1 and allele 2, respectively, based on the absolute VAF score (passed when VAF >= 40 for heterozygous and VAF >= 20 for homozygous);
calledPloidy: estimated ploidy (i.e. how many homologous copies of the genome are present); in absence of structural or copy number variation, it is expected to be 2 for autosomes, both in male and female, 2 for the X chromosome in female, and 1 for X and Y chromosomes in male (in the latter case, it is accompanied by “hap” zygosity); ploidy other than the expected value is either reflective of copy number or other structural variation, or presence of segmental duplications or other sequences with high mutual sequence similarity; when ploidy deviates from the expected value, called variants may be inaccurate.

5. Gene mapping and effect

typeseq: type of sequence overlapped, with respect to known genes/transcripts and their coding / noncoding status: (a) “exonic” represents coding exons, (b) “exonic:splicing” represents the beginning/end of coding exons which may also affect splicing, (c) “splicing” represents core splicing site (2 bp on the intron side of intron-exon and exon-intron junctions), (d) "ncRNA_exonic" represents exons of non-coding RNA genes, (e) "ncRNA_splicing" represents core splicing sites of non-coding RNA genes , (f) “UTR5” represents 5’ untranslated region, (g) “UTR3” represents 3’ unstranslated region, (h) “upstream” represents 1kb ubstream of TSS, (i) “downstream” represents 1kb downstream of TSS, and (j) “intergenic” represents intergenic regions (beyond the distance threshold of 1kb used for upstream/downstream);

refseq_id: combined Annovar output on coding sequence mapping and effect, composed of: (a) for coding-exonic changes (typeseq “exonic”): gene official symbol, RefSeq transcript isoform ID, coding sequence position and nucleotide change, amino acid sequence position and change (available only for missense variants); (b) for core splice site changes (typeseq "splicing") and non-coding sequence: gene official symbol.
effect: type of effect on the coding sequence: (a) “synonymous SNV”, (b) "nonsynonymous SNV" (actually representing missense SNV), (c) "stopgain SNV", (d) "frameshift deletion", (e) "frameshift insertion", (f) "frameshift substitution", (g) "nonframeshift deletion", (h) "nonframeshift insertion", (i) "nonframeshift substitution" , (j) “stoploss SNV”; effect is “NA” for typeseq == splicing;
aa_flag: this flag is set to 1 if more than one distinct amino acid change is reported in the refseq_id field;
gene_symbol: official gene symbol;
entrez.id: NCBI entrez-gene id;
gene_desc: full gene name;
gene_type: “protein-coding”, “snRNA” (small nuclear ncRNA), “snoRNA” (small nucleolar ncRNA), “tRNA”, “rRNA”, “ncRNA” (representing other types of ncRNA such as lincRNA, readthrough, antisense), “pseudo” (i.e. pseudogene), “unknown”;
omim_id: omim gene accession id;
omim_Phenotype: omim disorder/disease description when available for the corresponding omim morbidmap accession;
MPO: (set of) MPO (Mammalian Phenotype Ontology) [32] top level phenotype(s), imported from MGI (Mouse Genome Informatics, www.informatics.jax.org/) and mapped from the human orthologous of the mouse gene (based on NCBI Homologene); each top level phenotype associated to the gene is reported as: MPO term ID, MPO term description, type of experiment (“het” for heterozygous knock-out or other mutated allele, “hom” for homozygous knock-out or other mutated allele, “X” for X-linked, “TG” for transgene, “complex” for multi-genic), using “@” as separator; primary MGI MPO annotations are up-propagated to top level phenotypes using the MPO ontology graph; the type of experiment is up-propagated giving priority to het, X, and hom over other types (in that order);
HPO: (set of) HPO (Human Phenotype Ontology) [33] top level phenotype(s), imported from HPO and primarily supported by OMIM; each top level phenotype associated to the gene is reported as: HPO term ID, HPO term description, mode of inheritance (“AD” for autosomal dominant, “XL” for X-linked, “AR” for autosomal recessive), using “@” as separator; primary HPO annotations are up-propagated to top level phenotypes using the HPO ontology graph; modes of inheritance are up-propagated giving priority to AD over AR;
CGD_disease: (Mendelian) disorder(s) based on the Clinical Genomics Database [34] maintained by the NHGRI (National Human Genome Research Institute);
CGD_inheritance: Clinical Genomics Database mode of inheritance (“AD”, “AR”, “AD/AR”, “XL”, more complex and not standardized modes of inheritance);
Gene_HI: predicted genic haploinsufficiency; a gene with score > 0.2 has moderate probability of haploinsufficiency, while a gene with score > 0.4 is more likely to be haploinsufficient [35];
Gene_GI: percentile of the genic intolerance score; a gene with score < 10-20 is under negative selection for non-synonymous variation and thus could be a disease-implicated gene [36].

6. Allele frequencies

X1000g_all: allele frequency in the full 1000 Genome data-set [37];
X1000g_eur: allele frequency in the Caucasian European sub-set of 1000 Genome;
X1000g_amr: allele frequency in the admixed American (Mexicans, Puerto Ricans, Peruvians, Colombians) sub-set of 1000 Genome;
X1000g_asn: allele frequency in the East-Asian sub-set of 1000 Genome;
X1000g_afr: allele frequency in the African sub-set of 1000 Genome;
NHLBI_all**:** allele frequency in the full NHLBI-ESP data-set [8]
NHLBI_eu**:** allele frequency in the Caucasian European sub-set of NHLBI-ESP
NHLBI_aa**:** allele frequency in the African-american sub-set of NHLBI-ESP;
cg**:** allele frequency in the 54 unrelated subject of the Complete Genomics control diversity panel, no quality filter;
cg_filtered**:** allele frequency in the 54 unrelated subject of the Complete Genomics control diversity panel, restricting to variants passing default Complete Genomics quality filters;
cgW597_AllFreq**,** cgW597_CalledFreq**,** cgW597_11s**,** cgW597_Hs**,** cgW597_Ls: allele frequencies in the Complete Genomics high coverage wgs of the Wellderly population of predominantly Caucasian healthy elderly people (597 individuals); "AllFreq" is the allele frequency based on the theoretical allele total number (2 * number of individuals), as provided for all the allele frequency databases described above; "CalledFreq" is the allele frequency adjusted for no calls (the denominator is the total number of called alleles); "11s" is the number of individuals carrying homozygous genotype; "Hs" is the number of high quality alleles; "Ls" is the number of low quality alleles; for all these fields, the allele is matched to the alternate allele of the annotated variant;
cg1KG436_AllFreq**,** cg1KG436_CalledFreq**,** cg1KG436_11s**,** cg1KG436_Hs**,** cg1KG436_Ls: allele frequencies in the Complete Genomics high coverage wgs of 1000 Genomes subset (436 individuals); same frequency-related fields as for cgW597;

7. Variation databases

dbsnp**:** exact match (by coordinates, reference allele and alternate allele) to dbSNP;
dbsnp_common**:** exact match (by coordinates, reference allele and alternate allele) to the common dbSNP track from UCSC;
dbsnp_region: overlap-based match for dbSNP;
dbsnp_common_region: overlap-based match for to the common dbSNP track from UCSC;
dbsnp_wind: window (+/- 7 bp) overlap-based match for dbSNP;
cosmic**:** exact match (position, allele) to the Cosmic database of somatic variants;
Clinvar_SIG: clinvar significance code ("non-pathogenic": benign, "probable-non-pathogenic": likely benign, "unknown": uncertain significance, "untested": not provided in the original submission (includes the cases where data are not available or unknown), "probable-pathogenic": likely pathogenic, "pathogenic": pathogenic, "drug-response": drug response, "histocompatibility": histocompatibility);
Clinvar_CLNDBN: clinvar associated disorder/disease;

Clinvar_CLNACC: clinvar accession ID;
HGMD_Accession: HGMD variant ID;
HGMD_type: type of sequence code (1: coding, 2: splicing, 3: regulatory);
HGMD_tag: pathogenicity code ("DM": pathogenic, "DM?": potentially pathogenic, "DFP": disease-associated polymorphisms from GWAS with additional supporting functional evidence, "DP": disease-associated polymorphisms from GWAS, "FP": are in vitro / in vivo functional polymorphisms affecting the structure, function or expression of the gene product, but with no disease association reported yet, "FTV": polymorphic or rare variants reported in the literature and predicted to alter the gene product, but with no disease association reported yet, "R": retired record, because no longer be disease causing);
HGMD_Disease: genetic disorder(s) associated to the variant;
HGMD_PubmedId: PubMed IDs associated to the HGMD entry.

8. Impact prediction and conservation

sift_score**:** dbNSFP [38] pre-computed SIFT score for predicted protein impact (values <= 0.05 correspond to damaging) [39];
polyphen_score: dbNSFP pre-computed Polyphen2 HDIV scores for predicted protein impact, (values >= 0.95 correspond to damaging) [40];
ma_score: dbNSFP pre-computed Mutation Assessor (MA) scores for predicted protein impact (values >= 2 can be considered as corresponding to damaging) [41];
phylopPMam: single value or value array of PhyloP nucleotide-level conservation score(s) inferred from the Placental Mammal genome group (values >= 1 can be considered as moderate conservation, values >= 2.5 can be considered as strong conservation) [42];
phylopPMam_avg: average value of PhyloP nucleotide-level conservation score(s) inferred from the Placental Mammal genome group (values >= 1 can be considered as moderate conservation, values >= 2.5 can be considered as strong conservation);
phylopVert100: single value or value array of PhyloP nucleotide-level conservation score(s) inferred from the 100 Vertebrate genome group (values >= 1.5 can be considered as moderate conservation, values >= 4 can be considered as strong conservation);
phylopVert100_avg: average value of PhyloP nucleotide-level conservation score(s) inferred from the 100 Vertebrate genome group;
CADD_Raw: CADD variant deleteriousness score (SNV only), raw score [43];
CADD_phred: CADD variant deleteriousness score (SNV only), phred-transformed quantile (10 corresponds to the top 10% and 20 corresponds to the top 1%) [43];
phastCons_placental: PhastCons score for the Placental Mammal genome group, encoding conservation at the regional rather than nucleotide level [42];
pfam_annovar: overlap with coding sequence matching to a PFAM protein domain [44];
per_cds_affected: percentage of coding exonic sequence downstream the variant (important to further evaluate the effect of stopgain, frameshift and splicing variants);
per_transcripts_affected: percentage of transcripts with an exon overlapping the variant (important to further evaluate the effect of stopgain, frameshift and splicing variants);

9. Other annotations

SegDup: overlap with segmental duplications (UCSC Segmental Duplications track);
Repeat: UCSC RepeatMasker track overlap;
Rare_variants_per_gene: number of variants per gene that are: (i) high quality (using default Complete Genomics filters) and (ii) coding/splicing nonsynonymous and (iii) 5% rare;

10. Annotation tool and database versions

Tool and data-set versions:
- All databases are referred to hg19 genome build.
- Annovar: August 2013 version.
- Annovar database for 1000G: 2012 April version.
- Annovar database for NHLBI-ESP: esp6500si version (compiled 2013 January 22)
- Annovar databases for SIFT, PolyPhen2 HDIV, MutationAssessor: ljb23 (compiled 2014 February 22), based on dbNSFP.
- dbSNP: version 138.
- Clinvar: downloaded 2014 March 03,
- Cosmic: version 68.
- HGMD: licensed commercial version, downloaded February 2014.
- RefSeq: RefGene table, downloaded from UCSC 2013 February 12.
- OMIM: morbidmap downloaded 2013 October 17.
- CGD, HPO, MGI/MPO: downloaded and processed 2014 March 28.
- Annovar database for CADD: caddgt10 (compiled 2014 March 10).
- PhastCons: downloaded from UCSC 2013 November 8.
- SegDups: downloaded from UCSC 2011 October 25.
- PhyloP placental mammals: downloaded from UCSC 2013 February 22; PhyloP 100 vertebrates: downloaded from 2013 September 16.
- Repeats: downloaded from UCSC 2013 October 17.
- PFAM: downloaded from UCSC 2013 October 21.

SUPPLEMENTARY NOTE 7: SNV AND INDEL PRIORITIZATION

1. Prioritization strategy overview

Variants were prioritized considering these criteria:
- sequencing quality
- allele frequency (restricting to rare variants)
- predicted impact on coding or non-coding sequence
- pathogenic effect, as reported by disease variant databases
- zygosity and genic mode of inheritance
- disease and other abnormal phenotypes the gene is known to be implicated in

Two separate pipeline were utilized, following different strategies but based on overlapping criteria. The Primary pipeline ignores the variant classification by disease variant databases and

uniquely relies on quality, frequency (1%) and impact. The Secondary pipeline uses a more inclusive frequency filter (5%), places more importance on disease variant databases and reports variants only for genes implicated in human genetic disorders; it was originally designed to offer optimized performance for incidental or secondary findings. These pipelines were developed and tested as part of a broader initiative for whole genome sequencing of Mendelian disorders at the SickKids Centre for Applied Genomics (TCAG) and Department of Pediatric Laboratory Medicine (DPLM). More in detail:

A. Primary pipeline: the rarity filter is set at 1%, and only variants with a predicted damaging impact on genic coding sequence or genic non-coding RNA sequence are reported (including missense); UTR, intronic, and inter-genic variants are not reported; variants reported as "pathogenic" by the Clinvar and HGMD databases are not reported if they do not fit these criteria; variants are classified based on the zygosity and mode of inheritance, including genes not yet implicated in disease or abnormal phenotype;
B. Secondary pipeline: the rarity filter is set at 5%, and only variants (a) mapping to genes in CGD, and (b) with a clear loss-of-function effect (stopgain, frameshift, splicing) or classified as pathogenic by Clinvar or HGMD, are reported; this includes UTR, intronic and inter-genic variants, for which impact prediction is more difficult; variants are classified based on the zygosity and gene mode of inheritance.

These are the prioritized variant groups generated by the Primary pipeline:

- Homozygous (recessive)
- Potential compound-heterozygous (recessive)
- X-linked haploid
- Dominant

These are the prioritized variant groups generated by the Secondary pipeline:

- Autosomal dominant likely pathogenic
- X-linked likely pathogenic, male haploid
- X-linked likely pathogenic, female homozygous
- Complex likely pathogenic, homozygous or haploid
- Complex likely pathogenic, potential compound het
- Autosomal recessive likely pathogenic, homozygous
- Autosomal recessive likely pathogenic, potential compound het
- Complex, uncertain pathogenicity
- X-linked, carrier status
- Autosomal recessive, carrier status

2. Prioritization pipelines: definitions

A brief explanation is provided, together with a simplified R code snippet.

Sequencing quality tiers

Quality tier 1 excludes no calls, half calls (i.e. where only one allele could be called with

sufficient confidence), low quality calls, and is based on the default quality filters suggested by Complete Genomics. Quality tier 2 is based on stricter requirements for minimum sequencing depth and alternate allele support; ploidy other than 2 is allowed only for haploid variants (e.g. X chromosome, male) or when ploidy == 1 and zygosity == “hom” (in correspondence of losses). The quality tier is captured by the F_Qual field, with values {1, 2}.

High quality tier 1 definition:

```
(zygosity == "hap" & Allele1VarQuality == "PASS") |
(zygosity %in% c ("het-ref", "hom", "het-alt") &
      Allele1VarQuality == "PASS" & Allele2VarQuality == "PASS")
```

High quality tier 2 definition:

```
(totalReadCount >= 5 & (
(zygosity == "hap"     & Allele1VarQuality == "PASS" & calledPloidy == 1 &
      allele1ReadCount / totalReadCount >= 0.80) |
(zygosity == "het-ref" & Allele1VarQuality == "PASS" & Allele2VarQuality == "PASS" &
      calledPloidy == 2 & allele1VarScoreEAF >= 40 & allele2VarScoreEAF >= 40 &
      allele1ReadCount / totalReadCount >= 0.30) |
(zygosity == "het-alt" & Allele1VarQuality == "PASS" & Allele2VarQuality == "PASS" &
      calledPloidy == 2 & allele1VarScoreEAF >= 40 & allele2VarScoreEAF >= 40 &
      allele1ReadCount / totalReadCount >= 0.30 &
      allele2ReadCount / totalReadCount >= 0.30) |
(zygosity == "hom"     & Allele1VarQuality == "PASS" & Allele2VarQuality == "PASS" &
      calledPloidy == 2 & allele1VarScoreEAF >= 20 & allele2VarScoreEAF >= 20 &
      allele1ReadCount / totalReadCount >= 0.80) |
(zygosity == "hom"     & Allele1VarQuality == "PASS" & Allele2VarQuality == "PASS" &
      calledPloidy == 1 & allele1VarScoreEAF >= 20 & allele2VarScoreEAF >= 20 &
      allele1ReadCount / totalReadCount >= 0.80)) ))
```

Protein coding and ncRNA Type

Three categories of impacted sequence are defined:

- protein coding (coding exons or splicing)
- ncRNA (exons or splicing)
- other (UTR, intronic, inter-genic)

This is captured by the F_coding field, with values {“Coding”, “ncRNA”, “Other”}

Coding definition

```
typeseq %in% c ("exonic", "exonic;splicing", "splicing")
```

ncRNA definition

```
typeseq %in% c ("ncRNA_exonic", "ncRNA_splicing")
```

Other definition

```
! typeseq %in% c ("exonic", "exonic;splicing", "splicing", "ncRNA_exonic",
"ncRNA_splicing")
```

Allele Frequency (rarity tiers)

The rarity tier is used to represent that a variant does not exceed a set allele frequency for *any* of the frequency databases (1000 Genomes, NHLBI-ESP, ...). 1% is used for the Primary pipeline and 5% is used for the Secondary pipeline. The F_Rare field captures what is the rarity tier, with values {0.05, 0.01, 0}. Variants with frequency > 0.05 are not reported by either pipeline.

Definition of F_Rare == 0.01

```
(X1000g_all  <= 0.01) & (X1000g_eur  <= 0.01) &
(X1000g_amr  <= 0.01) & (X1000g_asn  <= 0.01) &
(X1000g_afr  <= 0.01) & (NLHBI_all   <= 0.01) &
(NLHBI_eu    <= 0.01) & (NLHBI_aa    <= 0.01) &
(cg          <= 0.01) & (cg_filtered <= 0.01) &
(cgW597_AllFreq <= 0.01) & (cg1KG436_AllFreq <= 0.01)
```

(replace the threshold values with 0.05 for F_Rare == 0.05):

Definition of F_Rare == 0

```
(X1000g_all  == 0) & (X1000g_eur  == 0) &
(X1000g_amr  == 0) & (X1000g_asn  == 0) &
(X1000g_afr  == 0) & (NLHBI_all   == 0) &
(NLHBI_eu    == 0) & (NLHBI_aa    == 0) &
(cg          == 0) & (cg_filtered == 0) &
(cgW597_AllFreq == 0) & (cg1KG436_AllFreq == 0) &
(is.na (dbsnp) & is.na (dbsnp_region))
```

Damaging

Damaging variants have some potential to significantly alter the gene product structure or expression regulation. They do not necessarily have a phenotypic outcome, as that depends also on properties that are typically captured at the gene level (e.g. a gene may be redundant, so that even a homozygous knock out does not produce a phenotype). Different definitions are used for protein coding and ncRNA.

Damage types (protein-coding):

- loss of function (“LOF”): stop-gain, splicing, frameshift, without any additional requirement
    - tier 2 (by default LOF corresponds to the highest damage tier)
- missense (“Missense”): missense with non-negative nucleotide-level conservation score AND at least one out of six predictive criteria met (high conservation PhyloP placental mammal, high conservation PhyloP 100-vertebrate, SIFT, Polyphen2, Mutation Assessor, CADD); thresholds for each predictive criterion were selected based on guidelines and/or to match the number of variants found to be damaging according to SIFT
    - tier 1: at least 1 of 6 predictive criteria (and non-negative conservation)
    - tier 2: at least 3 of 6 predictive criteria (and non-negative conservation)
- other coding (“OtherC”): stoploss, non-frameshift substitutions and in/dels with nucleotide-level conservation (PhyloP placental mammal or vertebrate) and no overlap with dbSNP;
    - tier 1 (by default OtherC cannot achieve tier 2)
- note that coding synonymous variants are never considered to be damaging, even in presence of very high conservation

Damage types (ncRNA):
- ncRNA (“DmgNcRNA”): nucleotide-level conservation (PhyloP placental mammal or vertebrate) AND regional conservation (phastCons placental mammal), OR CADD
    - tier 1: more inclusive conservation and CADD thresholds
    - tier 2: more stringent conservation and CADD thresholds

The F_DamageType field captures the damage type, with values = {“LOF”, “Missense”, “OtherC”, “DmgNcRNA”, “NO”}. The F_DamageTier field captures the damage tier, with values = {0, 1, 2}. 0 is used only for variants with F_DamageType = “NO”.

F_DamageType == “LOF” definition

```
F_Coding == "Coding" & (effect %in% eff_lof.chv | typeseq == "splicing")
```

F_DamageType == “Missense”, F_DamageTier == 1 definition

```
effect %in% "nonsynonymous SNV" & F_Coding == "Coding" &
	(phylopPMam_avg >= 0 | phylopVert100_avg >= 0) &
	((sift_score < 0.05) + (polyphen_score >= 0.95) + (ma_score >= 2.00) +
	(phylopPMam_avg >= 2.40) + (phylopVert100_avg >= 4.0) + (CADD_phred >= 15) >=
1)
```

F_DamageType == “Missense”, F_DamageTier == 2 definition

```
effect %in% "nonsynonymous SNV" & F_Coding == "Coding" &
	(phylopPMam_avg >= 0 | phylopVert100_avg >= 0) &
	((sift_score < 0.05) + (polyphen_score >= 0.95) + (ma_score >= 2.00) +
```

```
      (phylopPMam_avg >= 2.40) + (phylopVert100_avg >= 4.0) + (CADD_phred >= 15) >=
3)
```

F_DamageType == "OtherC", F_DamageTier == 1 definition

```
F_Coding == "Coding" & (
((effect %in% c ("nonframeshift deletion", "nonframeshift insertion",
      "nonframeshift substitution") &
      ((phylopPMam_avg >= 2.5 | phylopVert100_avg >= 4.0 | CADD_phred >= 20) &
      is.na (dbsnp_common) & is.na (dbsnp_common_region))) |
 (effect %in% ("stoploss SNV") &
      ((phylopPMam_avg >= 2.5 | phylopVert100_avg >= 4.0 | CADD_phred >= 20) &
      is.na (dbsnp_common) & is.na (dbsnp_common_region))) ) |
((effect %in% c ("nonframeshift deletion", "nonframeshift insertion",
      "nonframeshift substitution") &
      ((phylopPMam_avg >= 1.25 | phylopVert100_avg >= 2.0 | CADD_phred >= 15) &
      is.na (dbsnp) & is.na (dbsnp_region))) |
 (effect %in% ("stoploss SNV") &
      ((phylopPMam_avg >= 1.25 | phylopVert100_avg >= 2.0 | CADD_phred >= 15) &
      is.na (dbsnp) & is.na (dbsnp_region))) )
```

F_DamageType == "DmgNcRNA", F_DamageTier == 1 definition

```
F_Coding == "ncRNA" & (CADD_phred >= 15 |
((phylopPMam_avg >= 2.00 | phylopVert100_avg >= 3.50) & (! is.na
(phastCons_placental))))
```

F_DamageType == "DmgNcRNA", F_DamageTier == 2 definition

```
F_Coding == "ncRNA" & (CADD_phred >= 20 |
((phylopPMam_avg >= 2.50 | phylopVert100_avg >= 4.50) & (! is.na
(phastCons_placental))))
```

Gene mode of inheritance: dominant

HPO dominant: G_AXD_HPO == 1 definition

```
HPO %in% grep (HPO, pattern = "@AD|@XD", value = T)
```

CGD dominant: G_AXD_CGD == 1 definition

```
CGD_inheritance == "AD"
```

Gene Phenotype Tier

Tier 1 captures any gene with an associated phenotype in human or mouse; tier 2 captures

genes with a phenotype compatible with a pre-composed selection of disorder or abnomarmal phenotype implicated genes. The phenotype tier is captured by the field F_PhenoTier.

F_PhenoTier == 1 definition

```
(! is.na (omim_phenotype) & omim_phenotype != "") |
(! is.na (MPO) & MPO != "") |
(! is.na (HPO) & HPO != "") |
(! is.na (CGD_disease) & CGD_disease != "")
```

F_PhenoTier == 2 definition

```
entrez.id %in% gene_selection.eg
```

3. Prioritization output: primary pipeline

The prioritized variant tables include FM_[...] fields that identify different groups of prioritized variants. These are the prioritized variant groups generated by the Primary pipeline:

- Homozygous (recessive): high quality, rare, damaging, homozygous variants, regardless of the gene implication in disease / abnormal phenotype and relative mode of inheritance.
- Potential compound-heterozygous (recessive): sets of two (very rarely more) variants per gene that are high quality, rare, damaging; in absence of parents or short-range read-backed phasing, only “potential” compound hets can be found.
- X-linked haploid: high quality, rare, damaging, haploid X-linked variants, regardless of the gene implication in disease / abnormal phenotype and relative mode of inheritance
- Dominant: high quality, rare, damaging variant disrupting genes with dominant mode of inheritance; note that the dominant mode of inheritance is defined only for genes with a disease or abnormal phenotype in humans (HPO, CGD).

(Recessive) homozygous: FM_HOM == 1 definition

```
F_Rare <= 0.01 & F_DamageType != "NO" & zygosity == "hom"
```

(Recessive) potential compound-heterozygous

FM_PCHET == 2 definition

At least two variants satisfy the following requirement:

```
F_Rare <= 0.01 & F_DamageType != "NO" & F_Qual == 2
```

FM_PCHET == 1 definition

One of the two or both variants satisfy the following requirement, but not the previous

requirement:

```
F_Rare <= 0.01 & F_DamageType != "NO" & F_Qual >= 1
```

X-linked haploid: FM_XHAP == 1 definition

```
F_Rare <= 0.01 & F_DamageType != "NO" & zygosity == "hap" & chr == "X"
```

Dominant: FM_AXDOM == 1 definition

```
F_Rare <= 0.01 & F_DamageType != "NO" & (G_AXD_CGD == 1 | G_AXD_HPO == 1)
```

4. Prioritization output: secondary pipeline

The prioritized variant tables produced by the Secondary pipeline include FS[...]_[...] fields that identify different groups of prioritized variants:

- FS1_AD_Pathg_Any: Autosomal dominant likely pathogenic
- FS1_XL_Pathg_Hap: X-linked likely pathogenic, male haploid
- FS1_XL_Pathg_Hom: X-linked likely pathogenic, homozygous
- FS1_CX_Pathg_HomHap: Complex likely pathogenic, homozygous or haploid
- FS1_CX_Pathg_PotCompHet: Complex likely pathogenic, potential compound het
- FS1_AR_Pathg_Hom: Autosomal recessive likely pathogenic, homozygous
- FS1_AR_Pathg_PotCompHet: Autosomal recessive likely pathogenic, potential compound het
- FS1_CX_Uncertain: Complex, uncertain effect
- FS1_XL_Carrier: X-linked, carrier status
- FS1_AR_Carrier: Autosomal recessive, carrier status

Group definitions

```
FS1_Select == 1 & CGD_inheritance == "AD"

FS1_Select == 1 & CGD_inheritance == "XL" & zygosity == "hap" & chr == "X"

FS1_Select == 1 & CGD_inheritance == "XL" & zygosity == "hom" & chr == "X"

FS1_Select == 1 & (! CGD_inheritance %in% c ("AD", "AR", "XL")) &
(zygosity == "hom" | zygosity == "hap")

FS1_Select == 1 & (! CGD_inheritance %in% c ("AD", "AR", "XL")) & F_CmpHet_S1 >= 1

FS1_Select == 1 & CGD_inheritance == "AR" & zygosity == "hom"

FS1_Select == 1 & CGD_inheritance == "AR" & F_CmpHet_S1 >= 1
```

```
FS1_Select == 1 & (! CGD_inheritance %in% c ("AD", "AR", "XL")) &
zygosity %in% c ("het-ref", "het-alt") & F_CmpHet_S1 == 0

FS1_Select == 1 & CGD_inheritance == "XL" &
zygosity %in% c ("het-ref", "het-alt") & chr == "X"

FS1_Select == 1 & CGD_inheritance == "AR" &
zygosity %in% c ("het-ref", "het-alt") & F_CmpHet_S1 == 0
```

Selection tier definitions

Selection tiers represent how likely the selected variants are to be real and relevant, regardless of the mode of inheritance and pathogenic effect vs carrier status. In summary:

- tier 1: gene has disease in CGD AND loss-of-function (stop-gain, frameshift, splicing) OR ClinVar “pathogenic” or “probable-pathogenic” (but not ClinVar “not-pathogenic” or “probable-not-pathogenic”) OR HGMD_disease not null (any tag accepted)
- tier 2: gene has disease in CGD AND quality tier = 2 AND rarity tier <= 0.01 (i.e. frequency does not exceed 1% in any database) AND (loss-of-function (stop-gain, frameshift, splicing) OR ClinVar “pathogenic” or “probable-pathogenic” (but not ClinVar “not-pathogenic” or “probable-not-pathogenic”) OR HGMD_tag = DM (i.e. pathogenic))
- tier 3: gene has disease in CGD AND quality tier = 2 AND rarity tier <= 0.01 (i.e. frequency does not exceed 1% in any database) AND damaging AND (loss-of-function (stop-gain, frameshift, splicing) OR ClinVar “pathogenic” or “probable-pathogenic” (but not ClinVar “not-pathogenic” or “probable-not-pathogenic”) OR HGMD_tag = DM (i.e. pathogenic)

Tier 1 definition: FS1_Select == 1 definition

```
CGD_disease != "" &
       ((F_DamageType == "LOF") |
         (Clinvar_SIG %in% c ("pathogenic", "probable-pathogenic")) |
         (HGMD_Disease != ""))
```

Tier 2 definition: FS2_Select == 1 definition

```
CGD_disease != "" & F_Qual == 2 & F_Rare <= 0.01 & (
       (F_DamageType == "LOF") |
       (F_Clinvar_Pathg == 1) |
       (HGMD_tag == "DM" & F_Clinvar_notPathg == 0) )
```

Tier 3 definition: FS3_Select == 1 definition

```
CGD_disease != "" & F_Qual == 2 & F_Rare <= 0.01 & F_DamageType != "NO" & (
       (F_DamageType == "LOF") |
       (F_Clinvar_Pathg == 1) |
       (HGMD_tag == "DM" & F_Clinvar_notPathg == 0) )
```

Additional definitions

F_Clinvar_notPathg is set to 1 whenever at least one ClinVar record is reported as "non-pathogenic" or "probable-non-pathogenic".

```
grep (Clinvar_SIG, pattern = "non-pathogenic")
```

F_Clinvar_Pathg is set to 1 whenever at least one ClinVar record is reported as "pathogenic" or "probable-pathogenic", but not "non-pathogenic" or "probable-non-pathogenic".

```
setdiff (
grep (Clinvar_SIG, pattern = "pathogenic"),
grep (Clinvar_SIG, pattern = "non-pathogenic") )
```

5. Genes matching Roifman Syndrome phenotype spectrum for the phenotype tier 2

The gene selection used to identify phenotype tier-2 was the union of the genes annotated for growth disease/phenotype or immune disease/phenotype in human or mouse (i.e. HP:0001507, MP:0005378, HP:0002715, MP:0005387).

SUPPLEMENTARY REFERENCES

[1] Turunen JJ, Niemelä EH, Verma B, Frilander MJ. The significant other: splicing by the minor spliceosome. Wiley Interdiscip Rev RNA. 2013 Jan-Feb;4(1):61-76.

[2] Ashburner M, Ball CA, Blake JA, Botstein D, Butler H, Cherry JM, Davis AP, Dolinski K, Dwight SS, Eppig JT, Harris MA, Hill DP, Issel-Tarver L, Kasarskis A, Lewis S, Matese JC, Richardson JE, Ringwald M, Rubin GM, Sherlock G. Gene ontology: tool for the unification of biology. The Gene Ontology Consortium. Nat Genet. 2000 May;25(1):25-9.

[3] Kanehisa M, Goto S, Sato Y, Furumichi M, Tanabe M. KEGG for integration and interpretation of large-scale molecular data sets. Nucleic Acids Res. 2012 Jan;40(Database issue):D109-14.

[4] Croft D, Mundo AF, Haw R, Milacic M, Weiser J, Wu G, Caudy M, Garapati P, Gillespie M, Kamdar MR, Jassal B, Jupe S, Matthews L, May B, Palatnik S, Rothfels K, Shamovsky V, Song H, Williams M, Birney E, Hermjakob H, Stein L, D'Eustachio P. The Reactome pathway knowledgebase. Nucleic Acids Res. 2014 Jan;42(Database issue):D472-7.

[5] Schaefer CF, Anthony K, Krupa S, Buchoff J, Day M, Hannay T, Buetow KH. PID: the Pathway Interaction Database. Nucleic Acids Res. 2009 Jan;37(Database issue):D674-9.

[6] Shannon P, Markiel A, Ozier O, Baliga NS, Wang JT, Ramage D, Amin N, Schwikowski B, Ideker T. Cytoscape: a software environment for integrated models of biomolecular interaction networks. Genome Res. 2003 Nov;13(11):2498-504.

[7] Merico D, Isserlin R, Stueker O, Emili A, Bader GD. Enrichment map: a network-based method for gene-set enrichment visualization and interpretation. PLoS One. 2010 Nov 15;5(11):e13984.

[8] Tennessen JA, Bigham AW, O'Connor TD, Fu W, Kenny EE, Gravel S, McGee S, Do R, Liu X, Jun G, Kang HM, Jordan D, Leal SM, Gabriel S, Rieder MJ, Abecasis G, Altshuler D, Nickerson DA, Boerwinkle E, Sunyaev S, Bustamante CD, Bamshad MJ, Akey JM; Broad GO; Seattle GO; NHLBI Exome Sequencing Project. Evolution and functional impact of rare coding variation from deep sequencing of human exomes. Science. 2012 Jul 6;337(6090):64-9.

[9] Anders S, Huber W. Differential expression analysis for sequence count data. Genome Biol. 2010;11(10):R106.

[10] Trapnell C, Williams BA, Pertea G, Mortazavi A, Kwan G, van Baren MJ, Salzberg SL, Wold BJ, Pachter L. Transcript assembly and quantification by RNA-Seq reveals unannotated transcripts and isoform switching during cell differentiation. Nat Biotechnol. 2010 May;28(5):511-5.

[11] Mezlini AM, Smith EJ, Fiume M, Buske O, Savich GL, Shah S, Aparicio S, Chiang DY, Goldenberg A, Brudno M. iReckon: simultaneous isoform discovery and abundance estimation from RNA-seq data. Genome Res. 2013 Mar;23(3):519-29.

[12] He H, Liyanarachchi S, Akagi K, Nagy R, Li J, Dietrich RC, Li W, Sebastian N, Wen B, Xin B, Singh J, Yan P, Alder H, Haan E, Wieczorek D, Albrecht B, Puffenberger E, Wang H, Westman JA, Padgett RA, Symer DE, de la Chapelle A. Mutations in U4atac snRNA, a component of the minor spliceosome, in the developmental disorder MOPD I. Science. 2011 Apr 8;332(6026):238-40.

[13] Haan EA, Furness ME, Knowles S, Morris LL, Scott G, Svigos JM, Vigneswaren R. Osteodysplastic primordial dwarfism: report of a further case with manifestations similar to those of types I and III. Am J Med Genet. 1989 Jun;33(2):224-7.

[14] Edery P, Marcaillou C, Sahbatou M, Labalme A, Chastang J, Touraine R, Tubacher E, Senni F, Bober MB, Nampoothiri S, Jouk PS, Steichen E, Berland S, Toutain A, Wise CA, Sanlaville D, Rousseau F, Clerget-Darpoux F, Leutenegger AL. Association of TALS developmental disorder with defect in minor splicing component U4atac snRNA. Science. 2011 Apr 8;332(6026):240-3.

[15] Sigaudy S, Toutain A, Moncla A, Fredouille C, Bourlière B, Ayme S, Philip N. Microcephalic osteodysplastic primordial dwarfism Taybi-Linder type: report of four cases and review of the literature. Am J Med Genet. 1998 Oct 30;80(1):16-24.

[16] Nagy R, Wang H, Albrecht B, Wieczorek D, Gillessen-Kaesbach G, Haan E, Meinecke P, de la Chapelle A, Westman JA. Microcephalic osteodysplastic primordial dwarfism type I with biallelic mutations in the RNU4ATAC gene. Clin Genet. 2012 Aug;82(2):140-6.

[17] Abdel-Salam GM, Abdel-Hamid MS, Issa M, Magdy A, El-Kotoury A, Amr K. Expanding the phenotypic and mutational spectrum in microcephalic osteodysplastic primordial dwarfism type I. Am J Med Genet A. 2012 Jun;158A(6):1455-61.

[18] Abdel-Salam GM, Miyake N, Eid MM, Abdel-Hamid MS, Hassan NA, Eid OM, Effat LK, El-Badry TH, El-Kamah GY, El-Darouti M, Matsumoto N. A homozygous mutation in RNU4ATAC as a cause of microcephalic osteodysplastic primordial dwarfism type I (MOPD I) with associated pigmentary disorder. Am J Med Genet A. 2011 Nov;155A(11):2885-96.

[19] Klinge L, Schaper J, Wieczorek D, Voit T. Microlissencephaly in microcephalic osteodysplastic primordial dwarfism: a case report and review of the literature. Neuropediatrics. 2002 Dec;33(6):309-13.

[20] Kilic E, Yigit G, Utine GE, Wollnik B, Mihci E, Nur BG, Boduroglu K. A novel mutation in RNU4ATAC in a patient with microcephalic osteodysplastic primordial dwarfism type I. Am J Med Genet A. 2015 Apr;167A(4):919-21.

[21] Mandel K, Grunebaum E, Benson L. Noncompaction of the myocardium associated with Roifman syndrome. Cardiol Young. 2001 Mar;11(2):240-3.

[22] MacDonald JR, Ziman R, Yuen RK, Feuk L, Scherer SW. The Database of Genomic Variants: a curated collection of structural variation in the human genome. Nucleic Acids Res. 2014 Jan;42(Database issue):D986-92.

[23] Taybi H, Linder D. Congenital familial dwarfism with cephaloskeletal dysplasia. Radiology 89: 275-281, 1967.

[24] Thomas PS, Nevin NC. Congenital familial dwarfism with cephalo-skeletal dysplasia (Taybi-Linder syndrome). Ann. Radiol. 19: 187-192, 1976.

[25] Pierce MJ, Morse RP. The neurologic findings in Taybi–Linder syndrome (MOPD I/III): Case report and review of the literature. Am J Med Genet Part A 158A:606–610, 2012.

[26] Majewski F, Stoeckenius M, Kemperdick H. Studies of microcephalic primordial dwarfism III: an intrauterine dwarf with platyspondyly and anomalies of pelvis and clavicles--osteodysplastic primordial dwarfism type III. Am J Med Genet. 1982 May;12(1):37-42.

[27] Meinecke P, Schaefer E, Wiedemann HR. Microcephalic osteodysplastic primordial dwarfism: further evidence for identity of the so-called types I and III. Am J Med Genet. 1991 May 1;39(2):232-6.

[28] Majewski F. Caroline Crachami and the delineation of osteodysplastic primordial dwarfism type III, and autosomal recessive syndrome. Am J Med Genet. 1992 Sep 15;44(2):203-9.

[29] Argente J, Flores R, Gutiérrez-Arumí A, Verma B, Martos-Moreno GÁ, Cuscó I, Oghabian A, Chowen JA, Frilander MJ, Pérez-Jurado LA. Defective minor spliceosome mRNA processing results in isolated familial growth hormone deficiency. EMBO Mol Med. 2014 Mar;6(3):299-306.

[30] Will CL, Schneider C, Hossbach M, Urlaub H, Rauhut R, Elbashir S, Tuschl T, Lührmann R. The human 18S U11/U12 snRNP contains a set of novel proteins not found in the U2-dependent spliceosome. RNA. 2004 Jun;10(6):929-41.

[31] Turunen JJ, Will CL, Grote M, Lührmann R, Frilander MJ. The U11-48K protein contacts the 5' splice site of U12-type introns and the U11-59K protein. Mol Cell Biol. 2008 May;28(10):3548-60.

[32] Smith CL, Goldsmith CA, Eppig JT. The Mammalian Phenotype Ontology as a tool for annotating, analyzing and comparing phenotypic information. Genome Biol. 2005;6(1):R7.

[33] Robinson PN, Köhler S, Bauer S, Seelow D, Horn D, Mundlos S. The Human Phenotype Ontology: a tool for annotating and analyzing human hereditary disease. Am J Hum Genet. 2008 Nov;83(5):610-5.

[34] Solomon BD, Nguyen AD, Bear KA, Wolfsberg TG. Clinical genomic database. Proc Natl Acad Sci U S A. 2013 Jun 11;110(24):9851-5.

[35] Huang N, Lee I, Marcotte EM, Hurles ME. Characterising and predicting haploinsufficiency in the human genome. PLoS Genet. 2010 Oct 14;6(10):e1001154.

[36] Petrovski S, Wang Q, Heinzen EL, Allen AS, Goldstein DB. Genic intolerance to functional variation and the interpretation of personal genomes. PLoS Genet. 2013;9(8):e1003709.

[37] 1000 Genomes Project Consortium, Abecasis GR, Altshuler D, Auton A, Brooks LD, Durbin RM, Gibbs RA, Hurles ME, McVean GA. A map of human genome variation from population-scale sequencing. Nature. 2010 Oct 28;467(7319):1061-73.

[38] Liu X, Jian X, Boerwinkle E. dbNSFP v2.0: a database of human non-synonymous SNVs and their functional predictions and annotations. Hum Mutat. 2013 Sep;34(9):E2393-402.

[39] Ng PC, Henikoff S. Predicting deleterious amino acid substitutions. Genome Res. 2001 May;11(5):863-74.

[40] Adzhubei IA, Schmidt S, Peshkin L, Ramensky VE, Gerasimova A, Bork P, Kondrashov AS, Sunyaev SR. A method and server for predicting damaging missense mutations. Nat Methods. 2010 Apr;7(4):248-9.

[41] Reva B, Antipin Y, Sander C. Predicting the functional impact of protein mutations: application to cancer genomics. Nucleic Acids Res. 2011 Sep 1;39(17):e118

[42] Pollard KS, Hubisz MJ, Rosenbloom KR, Siepel A. Detection of nonneutral substitution rates on mammalian phylogenies. Genome Res. 2010 Jan;20(1):110-21.

[43] Kircher M, Witten DM, Jain P, O'Roak BJ, Cooper GM, Shendure J. A general framework for estimating the relative pathogenicity of human genetic variants. Nat Genet. 2014 Mar;46(3):310-5.

[44] Finn RD, Bateman A, Clements J, Coggill P, Eberhardt RY, Eddy SR, Heger A, Hetherington K, Holm L, Mistry J, Sonnhammer EL, Tate J, Punta M. Pfam: the protein families database. Nucleic Acids Res. 2014 Jan;42(Database issue):D222-30.
